# 3-Glucosylated 5-amino-1,2,4-oxadiazoles: synthesis and evaluation as glycogen phosphorylase inhibitors

**DOI:** 10.3762/bjoc.11.56

**Published:** 2015-04-17

**Authors:** Marion Donnier-Maréchal, David Goyard, Vincent Folliard, Tibor Docsa, Pal Gergely, Jean-Pierre Praly, Sébastien Vidal

**Affiliations:** 1 Institut de Chimie et Biochimie Moléculaires et Supramoléculaires (UMR 5246), Laboratoire de Chimie Organique 2, Université Claude Bernard Lyon 1 and CNRS; 43 Boulevard du 11 Novembre 1918, F-69622, Villeurbanne, France; 2 Department of Medical Chemistry, Faculty of Medicine, University of Debrecen, Egyetem tér 1, H-4032 Debrecen, Hungary

**Keywords:** amidoxime, carbodiimide, glycogen phosphorylase, oxadiazole, Vilsmeier salt

## Abstract

Glycogen phosporylase (GP) is a promising target for the control of glycaemia. The design of inhibitors binding at the catalytic site has been accomplished through various families of glucose-based derivatives such as oxadiazoles. Further elaboration of the oxadiazole aromatic aglycon moiety is now reported with 3-glucosyl-5-amino-1,2,4-oxadiazoles synthesized by condensation of a *C*-glucosyl amidoxime with *N*,*N*’-dialkylcarbodiimides or Vilsmeier salts. The 5-amino group introduced on the oxadiazole scaffold was expected to provide better inhibition of GP through potential additional interactions with the enzyme’s catalytic site; however, no inhibition was observed at 625 µM.

## Introduction

**Figure 1:**
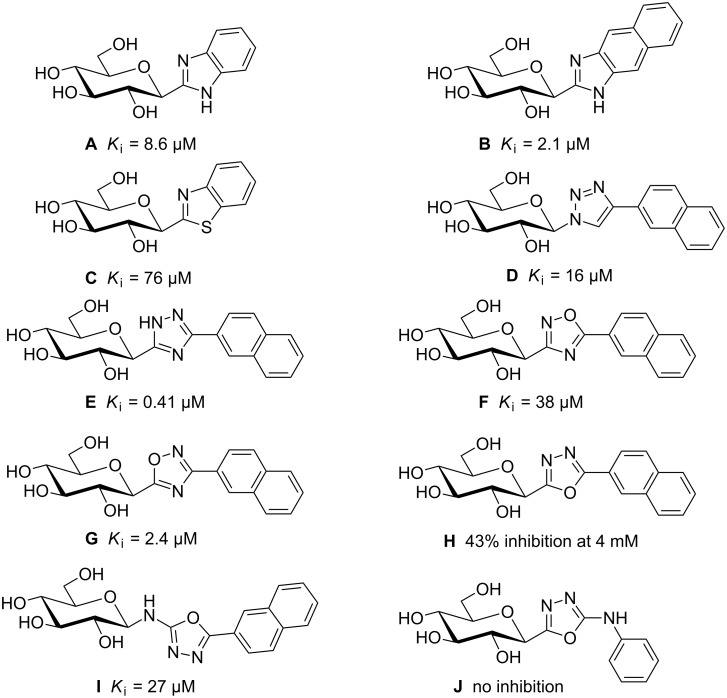
Structures of the glycosylated heterocyclic GP inhibitors and corresponding *K*_i_ values against RMGP*b*.

Glycogen phosphorylase (GP) is a homodimeric enzyme that is responsible for the depolymerization of glycogen into glucose-1-phosphate, which is further converted into glucose delivered into the blood stream [1–2]. The control of GP activity could find application in the treatment of hyperglycemia for type 2 diabetes patients [3–7]. Seven binding sites have been identified for GP and the design of various classes of inhibitors has attracted much attention [8–12]. *C*-Glycosylated heterocycles have been designed and synthesized and displayed low micromolar to even sub-micromolar *K*_i_ values against GP (Figure 1) [9–13]. Benzo-fused aglycons [14–15] (**A–C**) were initially identified as beneficial for the inhibition of GP. 1,2,3-Triazolyl-based aglycon **D** [16–17] proved valuable for the inhibition of GP and further design around the triazole moiety was also reported [18–19]. The isomeric 1,2,4-triazole **E** was recently identified as a sub-micromolar GP inhibitor [20–21] and highlights the influence of the aromatic moiety used in these studies. The influence of the heterocyclic moiety was also clearly demonstrated in a structure–activity relationship study with several isomeric oxadiazoles. The regioisomeric substitution around the 1,2,4-oxadiazoles (**F** [22–23] vs **G** [23–24]) plays a role in the inhibition observed with the glucosyl group at the 5-position of the 1,2,4-oxadiazole ring being preferred. Isomeric 1,3,4-oxadiazole **H** [25] was practically inactive against GP in comparison to its 1,2,4-oxadiazole congeners (**F** and **G**). Nevertheless, inhibition could be restored by introducing a nitrogen atom (NH) between the glucosyl and aromatic aglycon (**I** [26]), while introduction on the other position (**J** [27]) did not help.

**Figure 2:**
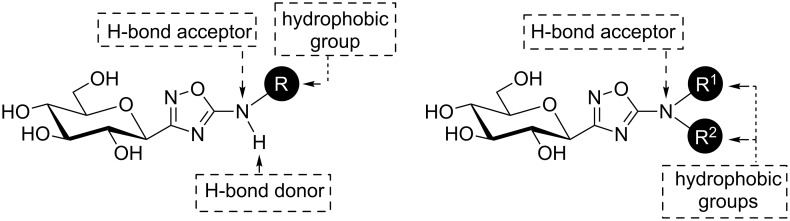
Structures of the two targeted 3-glucosyl-5-amino-1,2,4-oxadiazoles.

The structure–activity relationship of 5-aryl-1,2,4-oxadiazoles **F** and **G** towards GP inhibition highlighted a set of interactions of the aryl moieties with the enzyme’s β-channel [22–24]. The amino acids present in this empty pocket are of mixed character and can accommodate hydrophobic groups such as aryl moieties, with 2-naphthyl identified as the best pharmacophore for the series discussed above (Figure 1, **D–I**). Nevertheless, hydrogen bonds might also be created in this secondary binding site with other residues. The introduction of hydrogen bond donors and/or acceptors at the 5-position of the oxadiazole could provide better ligands for this enzyme through additional interactions with the β-channel amino acid residues (Figure 2). Nevertheless, introduction of a tetrahedral atom in the aglycon usually weakens the inhibitory properties [8–12]. The introduction of an amino functionality between the 1,3,4-oxadiazole core and glucose [26] (**I**) or aromatic [27] (**J**) groups was recently investigated (Figure 1). The present study reports on the introduction of such amino groups between the oxadiazole and aromatic cores. Two synthetic strategies were used to obtain such scaffolds based on recent and unprecedented literature reports.

## Results and Discussion

### Synthesis of 3-glucosylated 5-amino-1,2,4-oxadiazoles

**Scheme 1:**
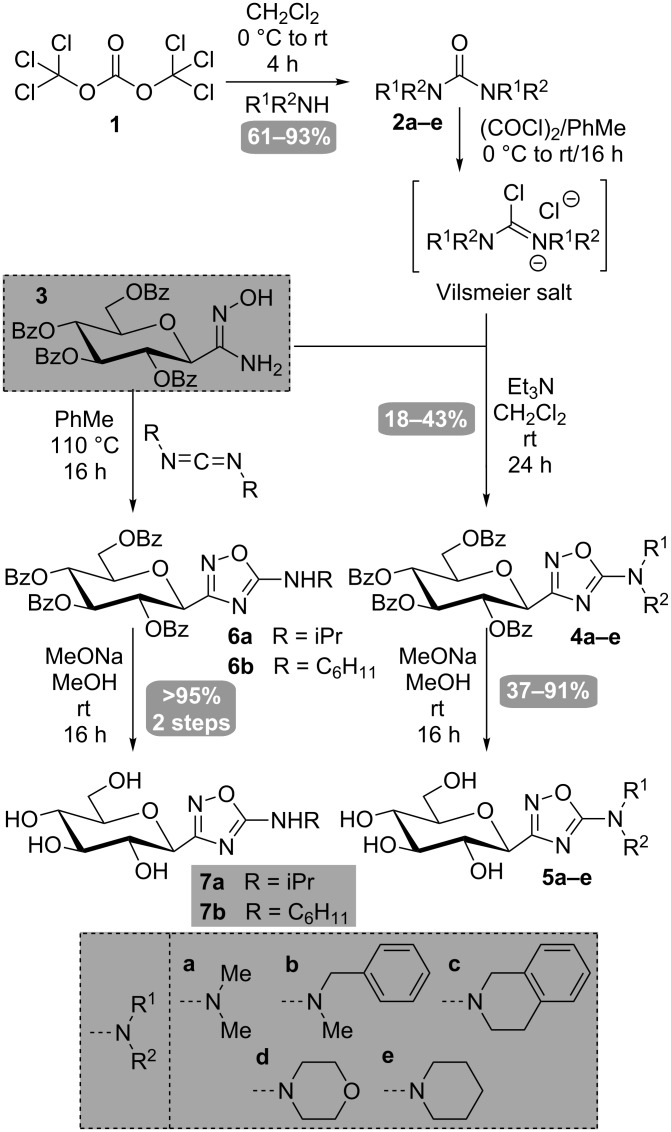
Synthesis of 3-glucosyl-5-amino-1,2,4-oxadiazoles.

The target GP inhibitor scaffold was obtained from the condensation of Vilsmeier salts with the *C*-glucosyl-amidoxime **3** [28] in the presence of a base. This condensation was very recently reported in the context of heterocyclic chemistry [29] and we have applied it with success to our GP inhibitor design. The dimethylamino group (Scheme 1, **a**) was chosen as a small pharmacophore that can be readily accommodated in the GP’s catalytic site. Aromatic moieties were also selected (Scheme 1, **b** and **c**) for their expected hydrophobic contact with the enzyme. The tetrahydroisoquinoline moiety (Scheme 1, **c**) was particularly interesting due to its structural analogy with the 2-naphthyl residue, which is identified as the best pharmacophore in the GP inhibitor series. The morpholinyl (Scheme 1, **d**) and piperidinyl (Scheme 1, **e**) residues were also selected as candidates for their hydrophobic properties, although they are bulkier than aromatic rings.

The synthesis started with commercially available ureas (**2a** and **2e**) or through the synthesis of such ureas. While the synthesis from carbonyl diimidazole was not successful [30], and since phosgene was not considered due to its hazardous reaction conditions, ureas **2b–d** could be readily prepared from triphosgene **1** (as a phosgene precursor) and the corresponding amines (Scheme 1). While treatment of triphosgene with the corresponding amine at room temperature in dichloromethane [31] was sufficient for ureas **2a,c–e**, heating (40 °C) and the addition of triethylamine [32] were required for condensation with *N*-methylbenzylamine to obtain urea **2b**. The Vilsmeier salt was then generated in situ with oxalyl chloride from the corresponding ureas and condensation with *C*-glucosyl-amidoxime **3** afforded the desired 3-glucosylated 5-amino-1,2,4-oxadiazoles **4a–e** in moderate yields. Even though these results are modest, they are in agreement with the yields reported in the literature [29] for such condensations. Removal of the benzoate esters provided the desired GP inhibitor candidates **5a–e**.

The syntheses of 3-glucosyl-5-alkylamino-1,2,4-oxadiazoles **7a,b** were readily achieved by condensation of the *C*-glucosyl amidoxime **3** [28] with *N*,*N’*-dialkylcarbodiimides under refluxing toluene (Scheme 1) [33–35]. To this end, condensation of amidoxime **3** [28] with commercially available carbodiimides (R = iPr, C_6_H_11_, tolyl) provided the desired 5-alkylaminooxadiazoles **6a,b** in quantitative yields and deprotection of the glucopyranose ring under Zemplén conditions afforded the *O*-unprotected GP inhibitor candidates **7a,b**. No reaction was observed with the *N*,*N*’-ditolylcarbodiimide, although the starting material was consumed. The mechanism proposed by Ispikoudi et al. [33] proposes the amine moiety as a leaving group during this process. Anilines (R = tolyl) are worse leaving groups than alkylamines (R = iPr, C_6_H_11_), thus providing a likely explanation regarding the non-reactivity observed for this substrate.

### Enzymatic inhibition studies (RMGP*b*)

The seven 3-glucosyl-5-amino-1,2,4-oxadiazoles **5a–e** and **7a,b** were evaluated as GP inhibitors using rabbit muscle glycogen phosphorylase *b* (RMGP*b*) as the model enzyme (see Supporting Information File 1 for details). The highest concentration used in the assay was 625 µM for compatibility of the DMSO stock solutions with the enzymatic reaction. No inhibition could be observed at this concentration.

This result compares unfavorably to the *C*-glucosylated heterocyclic inhibitors **A–F** (IC_50_ ~µM) [22–23]. These poor inhibitory properties could be due to limited interactions of alkyl groups in the β-channel of GP in comparison to aromatic moieties. This lack of GP inhibition for a 5-aminooxadiazole can be correlated with the lack of inhibition of compound **J** in the 1,3,4-oxadiazole series (Figure 1). While the introduction of an NH moiety between the anomeric carbon atom and the heteroaryl group was actually beneficial (compare **H** to **I** in Figure 1), the same NH moiety at the 5-position of the 1,3,4-oxadiazole ring was highly detrimental (compare **I** to **J** in Figure 1).

## Conclusion

In conclusion, we have synthesized seven 3-glucosyl-5-amino-1,2,4-oxadiazoles by condensation of a *C*-glucosyl amidoxime with Vilsmeier salts or carbodiimides. The synthetic strategy provided access to 5-alkylamino and 5-dialkylamino-1,2,4-oxadiazoles from the same glucose-based amidoxime precursor. Symmetric ureas were synthesized and converted into their Vilsmeier salts, which upon condensation with the amidoxime afforded the dialkylated 5-amino-1,2,4-oxadiazoles. Condensation of three commercially available carbodiimides led to two mono-alkylated 5-amino products, while the aromatic (i.e., tolyl) carbodiimide did not provide the desired product. A series of seven glucose-based inhibitors of GP were then evaluated but did not display any inhibition against RMGP*b* at 625 µM.

## Supporting Information

File 1 Experimental details and NMR data for all new compounds as well as enzyme kinetics (IC_50_) measurements.
