# Supplementary material for: 3-Glucosylated 5-amino-1,2,4-oxadiazoles: synthesis and evaluation as glycogen phosphorylase inhibitors
Source: Beilstein J Org Chem. 2015 Apr 17;11:499–503. doi: 10.3762/bjoc.11.56 (PMC4419504; doi:10.3762/bjoc.11.56)
Supplement: File 1 — Experimental details and NMR data for all new compounds as well as enzyme kinetics (IC50) measurements. [file Beilstein_J_Org_Chem-11-499-s001.pdf]

Supporting Information

for

3-Glucosylated 5-amino-1,2,4-oxadiazoles: synthesis and evaluation as glycogen phosphorylase inhibitors

Marion Donnier-Maréchal[1], David Goyard[1], Vincent Folliard[1], Tibor Docsa[2], Pal Gergely[2], Jean-Pierre Praly[1] and Sébastien Vidal*[1]

Address: [1]Institut de Chimie et Biochimie Moléculaires et Supramoléculaires (UMR 5246); Laboratoire de Chimie Organique 2, Université Claude Bernard Lyon 1 and CNRS; 43 Boulevard du 11 Novembre 1918, F-69622, Villeurbanne, France and [2]Department of Medical Chemistry, Faculty of Medicine, University of Debrecen, Egyetem tér 1, H-4032 Debrecen, Hungary

Email: Sébastien Vidal - sebastien.vidal@univ-lyon1.fr

*Corresponding author

Experimental details and NMR data for all new compounds as well as enzyme kinetics (IC_{50}) measurements

General methods and general procedures — **Pages** S2 to S3

Synthetic procedures, characterizations and NMR spectra — **Pages** S4 to S26

Enzymatic inhibition — **Page** S27

General methods. All reagents and solvents used for syntheses were commercial and used without further purification. Solvents were distilled over Mg/I_2 (MeOH), or purchased dry. Reactions were performed under argon atmosphere. NMR spectra were recorded at 293 K, unless stated otherwise, using a 300 MHz, a 400 MHz or a 500 MHz spectrometer. Shifts are referenced relative to deuterated solvent residual peaks. Low and high resolutions mass spectra were recorded using a Bruker MicrOTOF-Q II XL spectrometer. Thin-layer chromatography (TLC) was carried out on aluminum sheets coated with silica gel 60 F_{254} (Macherey-Nagel). TLC plates were inspected by UV light (λ = 254 nm) and developed by treatment with a solution of 10% H_2SO_4 in EtOH/H_2O (1:1 v/v) followed by heating. Silica gel column chromatography was performed with silica gel Si 60 (40–63 μm). Thick-layer chromatography was performed to purify compounds on a 20–50 mg scale using standard silica coated TLC plates (thickness 200 μm) presented above. The compound was desorbed from the silica gel with CH_2Cl_2/MeOH 9:1. Optical rotations were measured using a Perkin Elmer polarimeter and values are given in 10^{-1} deg·cm^2·g^{-1}. Melting points were measured on a Büchi apparatus and are uncorrected. Ureas **1a** and **1e** were purchased from commercial sources.

General procedure A for the synthesis of urea (2b–d):

To a solution of amine (5–10 equiv) in anhydrous dichloromethane (25 mL) at 0 °C was added dropwise a solution of triphosgene (1 equiv) in anhydrous dichloromethane (5 mL). The reacting mixture was stirred at 0 °C for 1 h and then stirred at room temperature for additional 4 h. After completion, water (100 mL) was added to dissolve the precipitate. The aqueous layer was extracted with dichloromethane (2 × 25 mL). The combined organic layers were washed with 1 N HCl (25 mL), saturated $NaHCO_3$ (25 mL) and brine (25 mL). The solution was dried (Na_2SO_4) and concentrated to give the desired product of satisfying purity (>95%) for use in the next step.

General procedure B for the synthesis of 3-(tetra-*O*-benzoyl-β-D-glucopyranosyl)-5-dialkylamino-1,2,4-oxadiazoles (4a–e):

Oxalyl chloride (1.2 equiv) was added dropwise to a solution of urea **2a–e** (1 equiv) in anhydrous toluene or anhydrous dichloromethane (10 mL) at 0 °C. The resulting mixture was stirred at room temperature overnight. After completion, the solvent was removed under vacuum and the solid was washed with cold ethyl acetate (3 × 5 mL) to give the desired Vilsmeier salt intermediate of sufficient purity for use in the next step.

A solution of 3-(2,3,4,6-tetra-*O*-benzoyl-β-D-glucopyranosyl)-formamidoxime **3** (1 equiv) and triethylamine (4 equiv) in anhydrous dichloromethane (10 mL) was added dropwise to a solution of the freshly prepared Vilsmeier salt in anhydrous dichloromethane (10 mL). The resulting mixture was stirred at room temperature overnight. After completion, the solvent was evaporated under vacuum and the crude product purified by silica gel column chromatography to afford compounds **4a–e**.

General procedure C for debenzoylation (5a–e and 7a,b):

Benzoylated compound **4a–e** and **6a,b** was dissolved in methanol/dichloromethane (3:1) and sodium methoxide was added until pH reached 9. The resulting mixture was stirred overnight at room temperature. After completion, the crude product was purified by silica gel column chromatography or thick layer chromatography.

General procedure D for the synthesis of 3-(β-D-glucopyranosyl)-5-alkylamino-1,2,4-oxadiazoles (6a,b):

A solution of *C*-(2,3,4,6-tetra-*O*-benzoyl-β-D-glucopyranosyl)-formamidoxime **3** (100 mg, 0.16 mmol, 1 equiv) and *N*,*N'*-dialkylcarbodiimide (0.33 mmol, 2.1 equiv) in toluene (2 mL) was stirred at 110 °C for 16 h and concentrated in vacuo. The crude product was purified by silica gel column chromatography (PE/EtOAc 6:4) to afford 3-(2,3,4,6-tetra-*O*-benzoyl-β-D-glucopyranosyl)-5-alkylamino-1,2,4-oxadiazole **6a,b** with a small portion of urea derived from the carbodiimide. The benzoylated compounds **6a,b** were deacetylated using standard Zemplén conditions (NaOMe in MeOH) and purified by silica gel chromatography (CH_2Cl_2/MeOH 9:1) to afford the 3-(β-D-glucopyranosyl)-5-alkylamino-1,2,4-oxadiazoles **7a,b**.

_N,N'_-dimethyl-_N,N'_-dibenzylurea (2b)

Obtained as yellow oil (1.26 g, 4.1mmol, 61%) following the general procedure A: *N*-methylbenzylamine (2 mL, 15.4 mmol), triphosgene (919 mg, 3.1 mmol) and triethylamine (2.1 mL, 15.4 mmol).

R_f = 0.57 (CH_2Cl_2); **^{1}H NMR ($CDCl_3$, 300 MHz)** δ 7.25-7.14 (m, 10H, H-arom), 4.33 (s, 4H, CH_2), 2.69 (s, 6H, CH_3); **^{13}C NMR ($CDCl_3$, 75 MHz)** δ 165.1 (C=O), 137.7 (C^{IV}-arom), 128.6 (CH-arom), 127.7 (CH-arom), 127.3 (CH-arom), 54.4 (CH_2), 36.7 (CH_3); **HR-ESI-QTof** *m/z* $[M+H]^+$ calcd. for $C_{17}H_{21}N_2O_1$ 269.1648; found 269.1646.

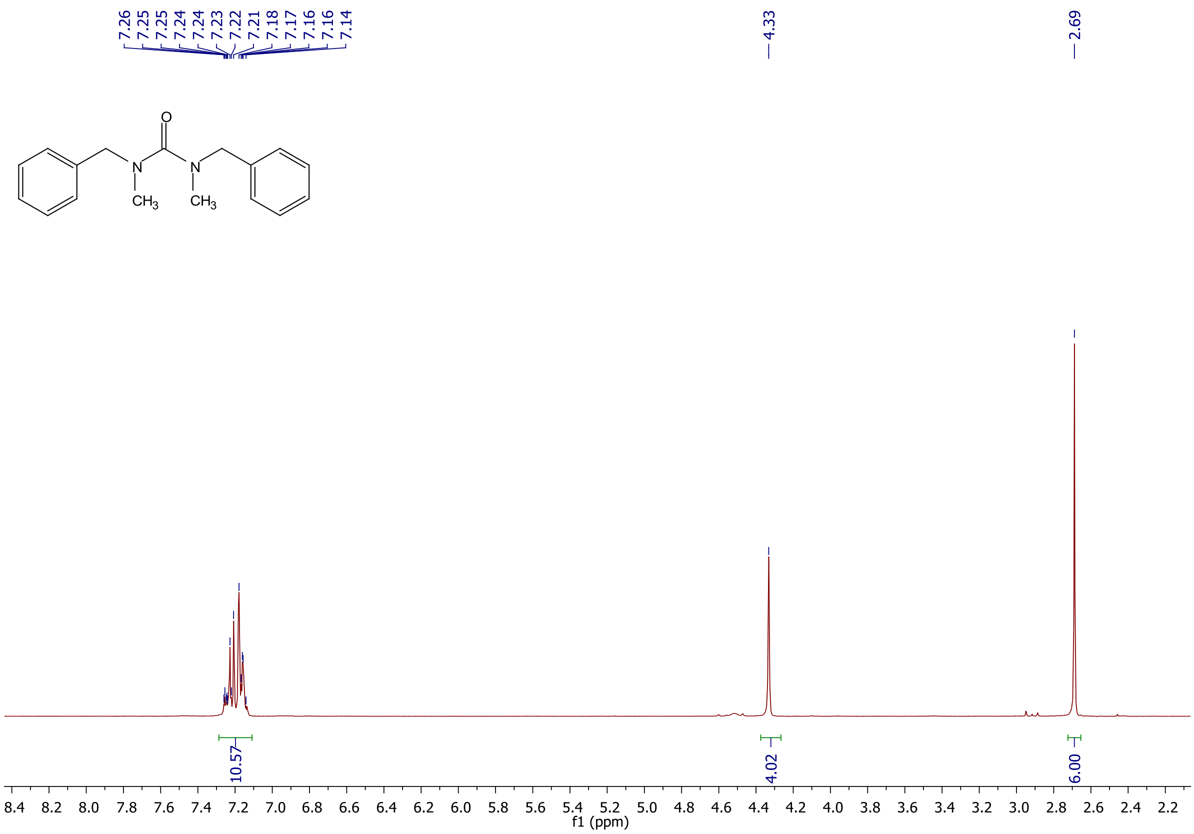

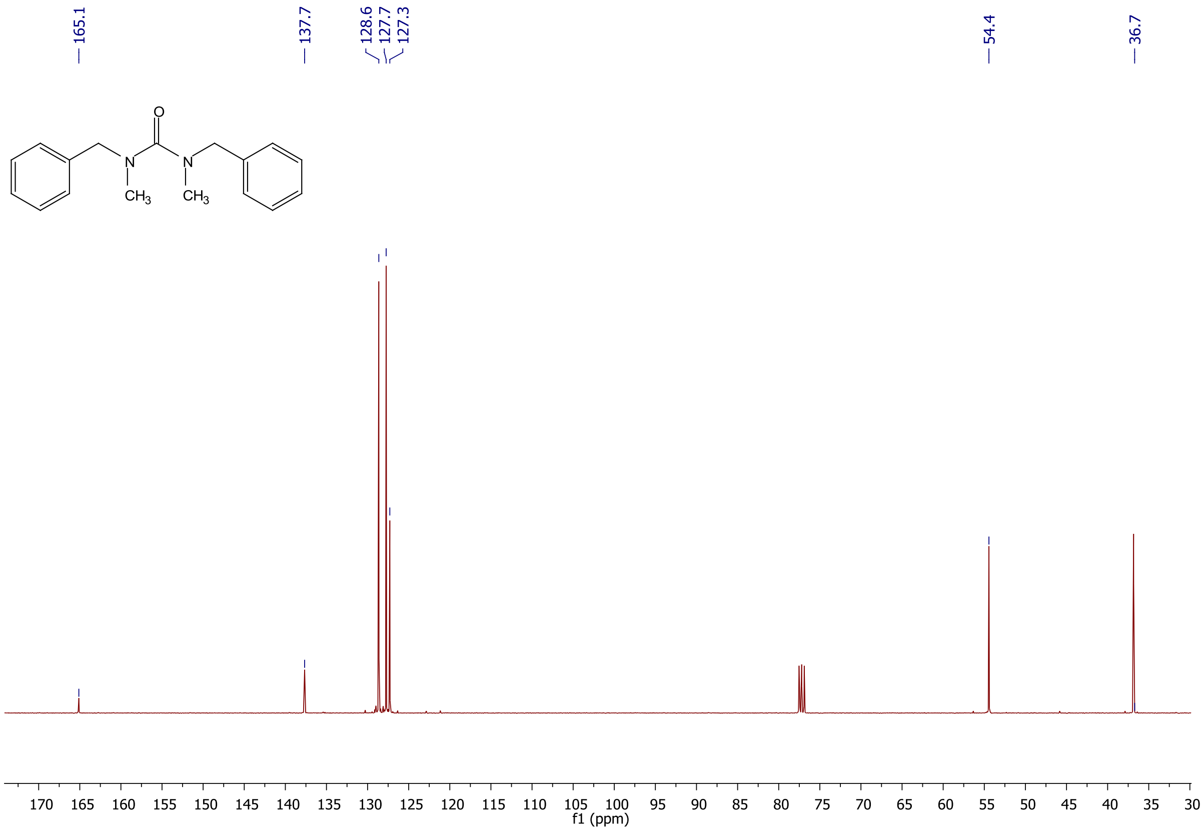

165.1
137.7
128.6
127.7
127.3
54.4
36.7
O
N
N
CH3
CH3
170 165 160 155 150 145 140 135 130 125 120 115 110 105 100 95 90 85 80 75 70 65 60 55 50 45 40 35 30
f1 (ppm)

2,2'-Carbonyl-di-(1,2,3,4-tetrahydroisoquinoline) (2c) [*J. Org.Chem.* **2003**, *68*, 7289-7297] Obtained as white powder (832 mg, 2.87 mmol, 65%) following the general procedure A: 1,2,3,4-tetrahydroisoquinoline (4 mL, 32.0 mmol), triphosgene (1.3 g, 4.38 mmol). The crude product necessitated a recrystallization from ethanol-diethyl ether.

^{1}H NMR ($CDCl_3$, 300 MHz) δ 7.21-7.09 (m, 8H, H-arom), 4.51 (s, 4H, NC$\underline{H}_2$), 3.57 (t, 4H, *J* = 5.8 Hz, NC$\underline{H}_2$$CH_2$), 2.96 (t, 4H, *J* = 5.8 Hz, NCH_2C$\underline{H}_2$).

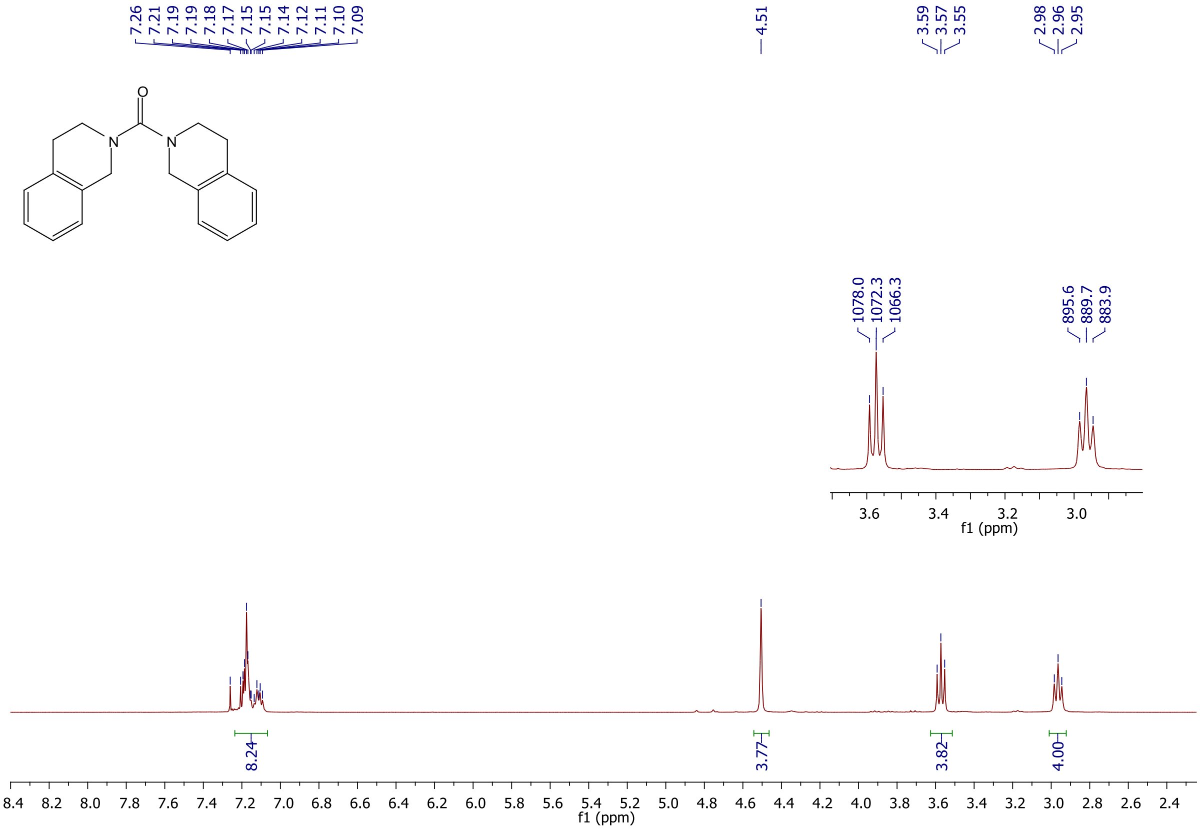

4,4'-Carbonyl-dimorpholine (2d) [*Synthesis* **2010**, 4251-4255]

Obtained as white powder (1.0 g, 5.02 mmol, 93%) following general procedure A: morpholine (4.70 g, 54 mmol), triphosgene (1.60 g, 5.40 mmol) in anhydrous dichloromethane (25 mL).

^{1}H NMR ($CDCl_3$, 300 MHz) δ 3.66 (t, 4H, *J* = 4.8 Hz, $NC\underline{H}_2CH_2$), 3.25 (t, 4H, *J* = 4.8 Hz, $NCH_2C\underline{H}_2$).

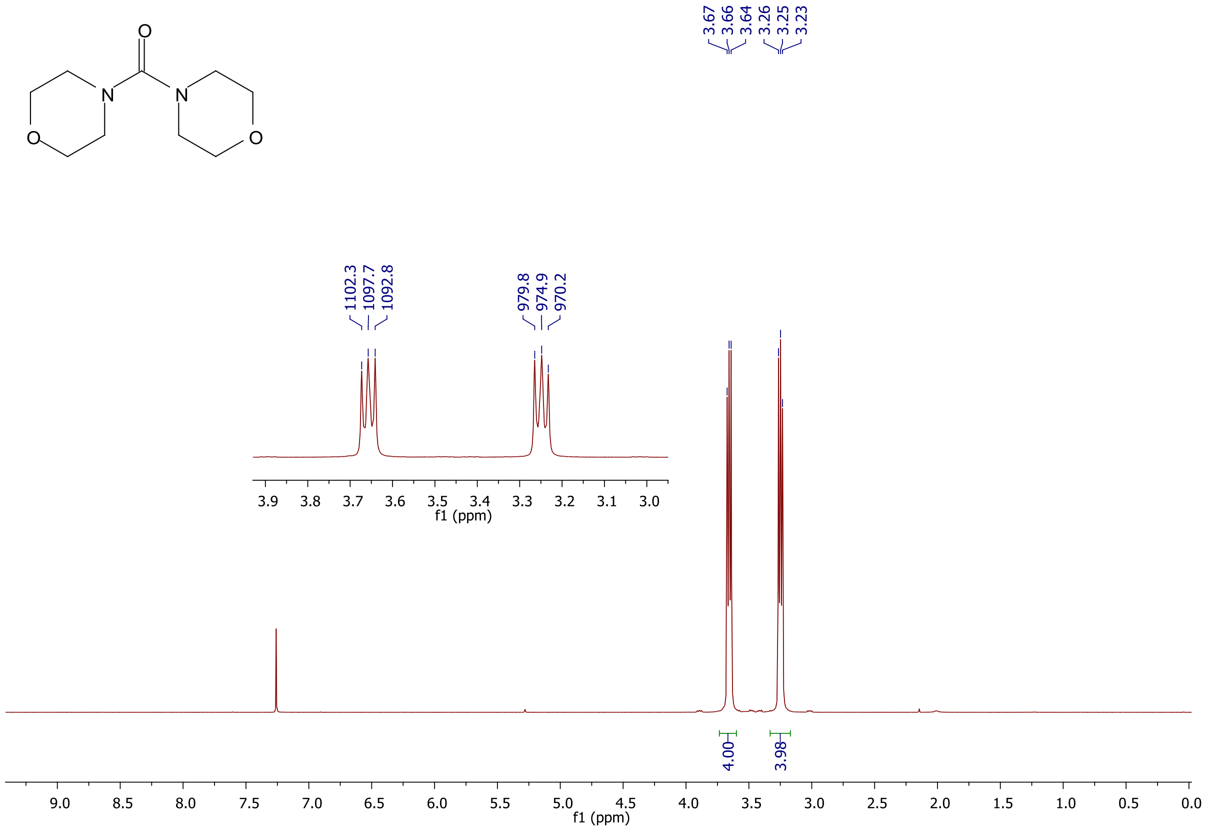

3-(2,3,4,6-Tetra-*O*-benzoyl-β-D-glucopyranosyl)-5-(*N*,*N*-dimethylamino)-1,2,4-oxadiazole (4a)

Obtained as white foam (230 mg, 0.33 mmol, 43%) following general procedure B: *N*-[chloro(dimethylamino)methylene)-*N*-methylmethanaminium chloride (260 mg, 1.57 mmol), 3-(2,3,4,6-tetra-*O*-benzoyl-β-D-glucopyranosyl)-formamidoxime **3** (500 mg, 0.78 mmol), triethylamine (435 μL, 3.12 mmol) and purified by silica gel column chromatography (EtOAc/PE, 35/65 to 40/60, v/v).

R_f = 0.3 (EtOAc/PE, 40/60, v/v); $[\alpha]_D^{20}$ = +6 (c 1.1, CH_2Cl_2); **^{1}H NMR ($CDCl_3$, 400 MHz)** δ 8.13-7.77 (m, 8H, H-arom), 7.60-7.20 (m, 12H, H-arom), 6.05-5.93 (m, 2H, H-3 and H-2), 5.82 (t, 1H, *J* = 9.6 Hz, H-4), 4.87 (d, 1H, *J* = 9.1 Hz, H-1), 4.64-4.60 (m, 1H, H-6b), 4.54 (dd, 1H, *J* = 12.6 Hz, *J* = 4.5 Hz, H-6a), 4.31-4.27 (m, 1H, H-5), 3.07 (s, 6H, NCH_3); **^{13}C NMR ($CDCl_3$, 100 MHz)** δ 172.0 (C-5oxa), 166.7 (C-3oxa), 166.2 (C=O), 165.9 (C=O), 165.2, (C=O), 164.7 (C=O), 133.5 (C^{IV}-arom), 133.21 (C^{IV}-arom), 133.20 (C^{IV}-arom), 133.08 (C^{IV}-arom), 129.9-128.3 (m, CH-arom), 76.9 (C-5), 74.5 (C-3), 72.8 (C-1), 70.4 (C-2), 69.4 (C-4), 63.4 (C-6), 38.0 (NCH_3); **HR-ESI-QTof** *m/z* $[M+H]^+$ calcd. for $C_{38}H_{34}N_3O_{10}$ 692.2239; found 692.2256.

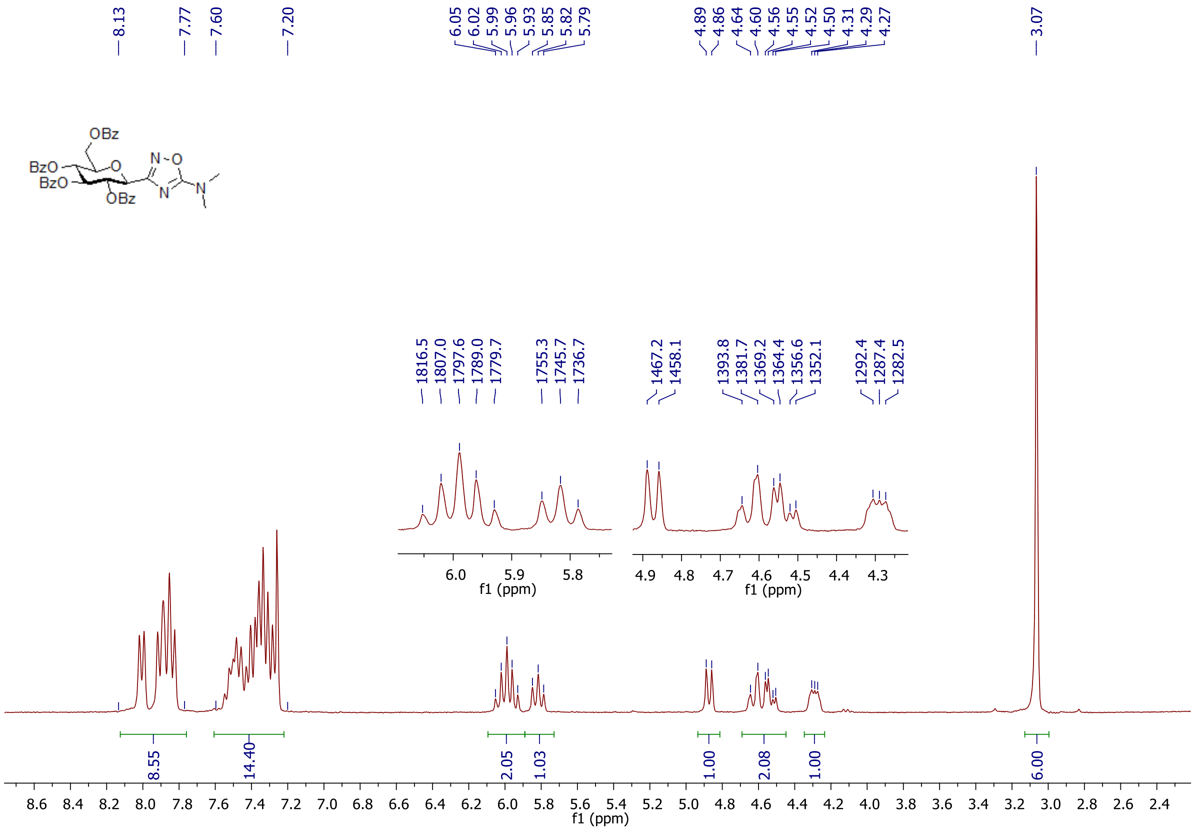

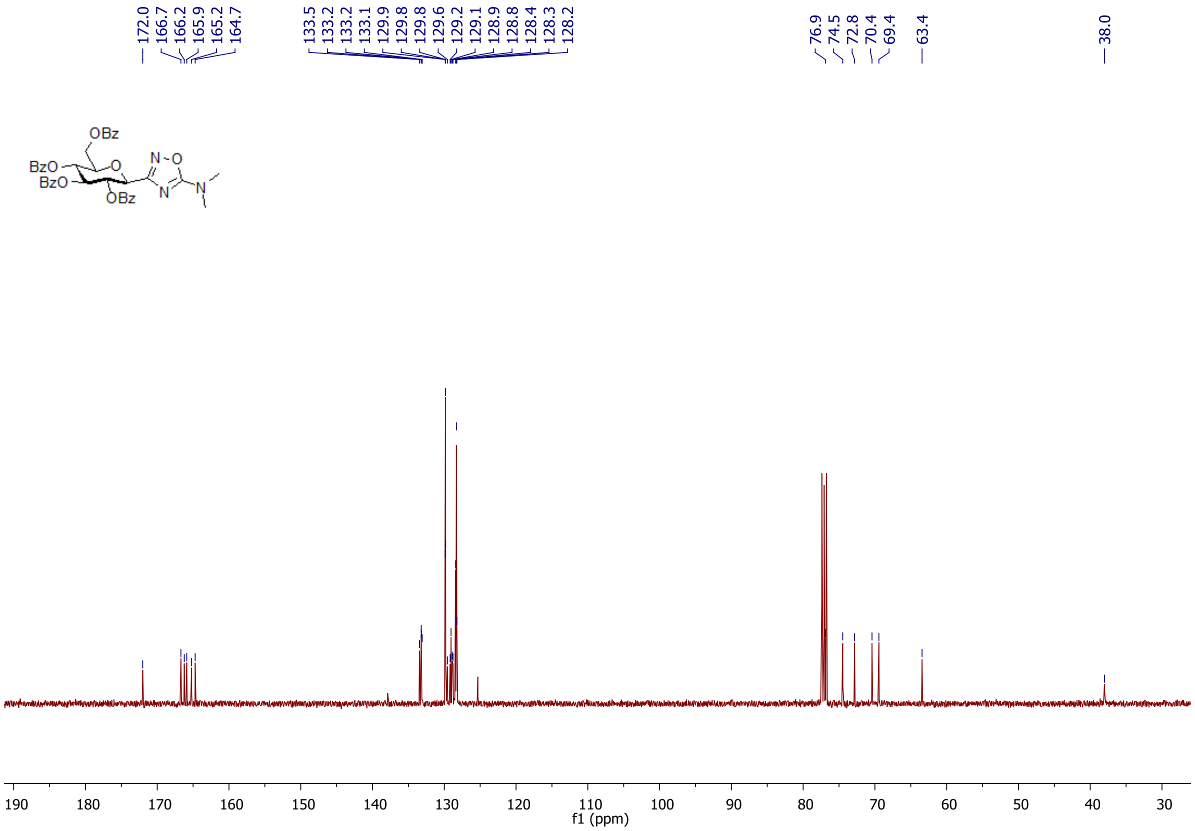

3-(2,3,4,6-Tetra-*O*-benzoyl-β-D-glucopyranosyl)-5-(*N*-methyl-*N*-benzylamino)-1,2,4-oxadiazole (4b)

Obtained as yellow oil (57 mg, 0.07 mmol, 24%) following general procedure B: *N*-[chloro(benzylmethylamino)methylene)-*N*-methylphenylmethanaminium chloride (200 mg, 0.62 mmol), 3-(2,3,4,6-tetra-*O*-benzoyl-β-D-glucopyranosyl)-formamidoxime **3** (197 mg, 0.31 mmol), triethylamine (173 µL, 1.24 mmol) andand purified by silica gel column chromatography (PE/ACOEt/MeOH, 70/24/6, v/v).

R_f = 0.44 (PE/AcOEt/MeOH, 70/24/6, v/v); $[\alpha]_D^{25}$ = -14 (c 0.3, CH_3OH); **^{1}H NMR ($CDCl_3$, 400 MHz)** δ 8.03-7.84 (m, 8H, H-arom), 7.55-7.24 (m, 15H, H-arom), 7.20-7.17 (m, 2H, H-arom), 6.06 (t, 1H,*J* = 9.6 Hz, H-3), 5.99 (t, 1H, *J* = 9.5 Hz, H-2), 5.83 (t, 1H, *J* = 9.9 Hz, H-4), 4.91 (d, 1H, *J* = 9.7 Hz, H-1), 4.72 (d, 1H, *J* = 15.2 Hz, C$\underline{H}_2$Ph), 4.64 (dd, 1H, *J* = 12.4 Hz, *J* = 2.8 Hz, H-6b), 4.54 (dd, 1H, *J* = 12.3 Hz, *J* = 5.0 Hz, H-6a), 4.44 (d, 1H, *J* = 15.2 Hz, C$\underline{H}_2$Ph), 4.33-4.29 (m,1H, H-5), 2.96 (s, 3H, NCH_3); **^{13}C NMR ($CDCl_3$, 100 MHz)** δ 172.2 (C-5oxa), 166.8 (C-3oxa), 166.2 (C=O), 165.9 (C=O), 165.2, (C=O), 164.7 (C=O), 135.3 (C^{IV}-arom), 133.4 (C^{IV}-arom), 133.2 (C^{IV}-arom), 133.1 (C^{IV}-arom),129.9-128.0 (m, CH-arom), 76.9 (C-5), 74.4 (C-3), 72.8 (C-1), 70.5 (C-2), 69.5 (C-4), 63.4 (C-6), 54.7 ($\underline{C}H_2$Ph), 35.1 (NCH_3); **HR-ESI-QTof** *m*/*z* $[M+Na]^+$ calcd. for $C_{44}H_{37}N_3NaO_{10}$ 790.2371; found 790.2335.

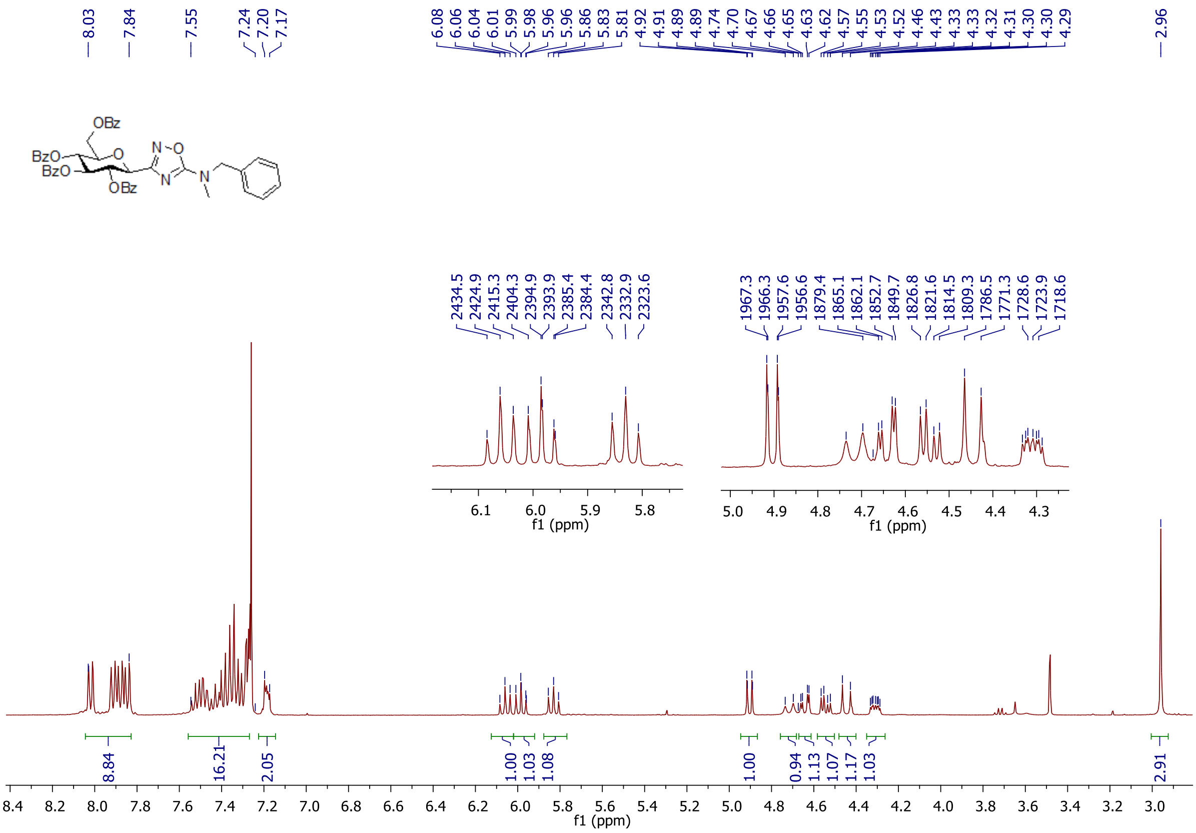

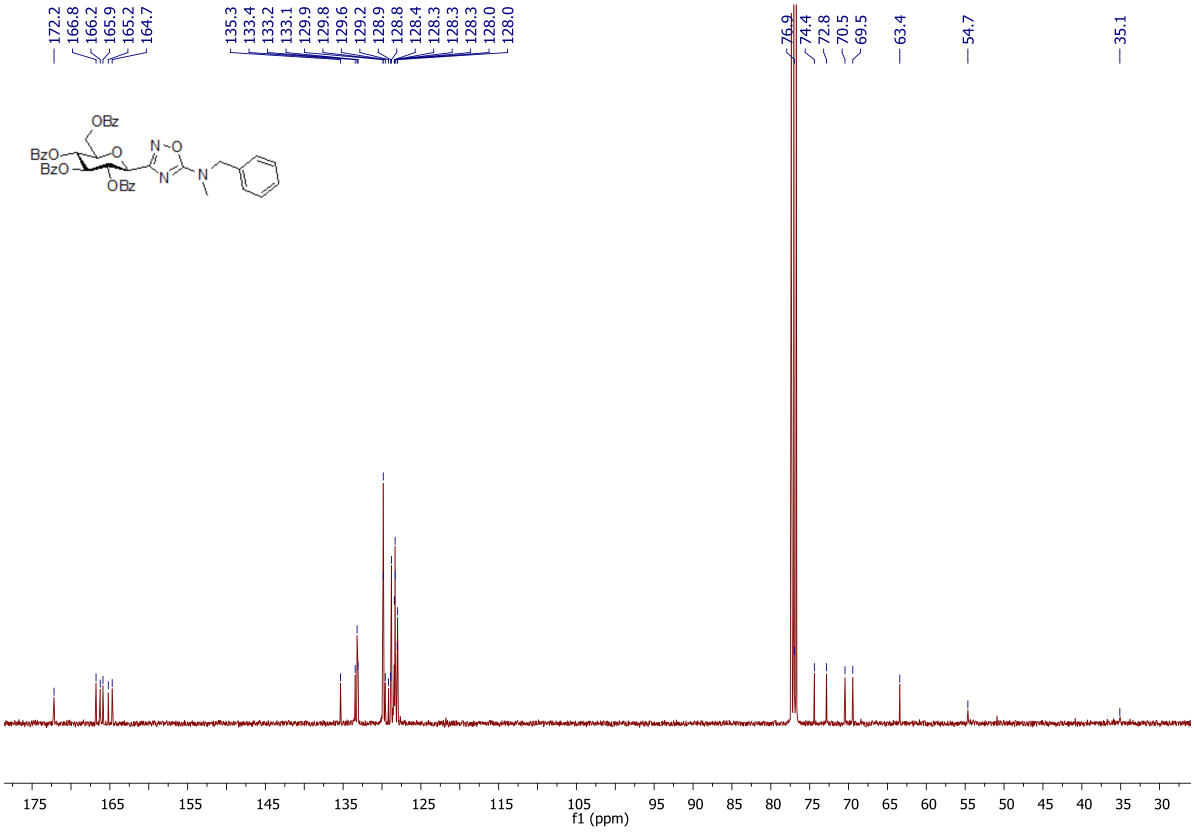

3-(2,3,4,6-Tetra-*O*-benzoyl-β-D-glucopyranosyl)-5-(1,2,3,4-tetrahydroisoquinolin-2-yl)-1,2,4-oxadiazole (4c)

Obtained as white foam (53 mg, 0.068 mmol, 18%) following general procedure B: 2-[chloro(1,2,3,4-tetrahydroisoquinolino)methylene]-1,2,3,4-tetrahydroisoquinolin-2-ium chloride, (282 mg, 0.82 mmol), 3-(2,3,4,6-tetra-*O*-benzoyl-β-D-glucopyranosyl)-formamidoxime **3** (236 mg, 0.37 mmol), triethylamine (206 µL, 1.48 mmol)and purified by silica gel column chromatography (EtOAc/PE, 35/65 to 40/60, v/v).

R_f = 0.3 (EtOAc/PE, 40/60, v/v); $[\alpha]_D^{20}$ = +24 (c 0.4, CH_2Cl_2); **^{1}H NMR ($CDCl_3$, 400 MHz)** δ 8.05-7.79 (m, 8H, H-arom), 7.58-7.26 (m, 12H, H-arom), 7.23-7.04 (m, 4H, H-arom), 6.06-5.96 (m, 2H, H-3 and H-2), 5.83 (t, 1H, *J* = 9.6 Hz, H-4), 4.90 (d, 1H, *J* = 9.5 Hz, H-1), 4.68 (s, 2H, $NC\underline{H}_2$), 4.62 (dd, 1H, *J* = 12.3 Hz, *J* = 3.0 Hz, H-6b), 4.54 (dd, 1H, *J* = 12.4 Hz, *J* = 5.2 Hz, H-6a), 4.32-4.29 (m, 1H, H-5), 3.80-3.77 (m, 2H, $NC\underline{H}_2$), 2.89 (t, 2H, *J* = 5.9 Hz, $C\underline{H}_2O$); **^{13}C NMR ($CDCl_3$, 100 MHz)** δ 171.1 (C-5oxa), 166.6 (C-3oxa), 166.2 (C=O), 165.9 (C=O), 165.2, (C=O), 164.7 (C=O), 133.7 (C^{IV}-arom), 133.5 (C^{IV}-arom), 133.22 (C^{IV}-arom), 133.20 (C^{IV}-arom), 133.1 (C^{IV}-arom), 131.5 (C^{IV}-arom), 129.9-128.3 (m, CH-arom), 127.0 (CH-arom), 126.7 (CH-arom), 126.3 (CH-arom), 76.9 (C-5), 74.4 (C-3), 72.9 (C-1), 70.4 (C-2), 69.4 (C-4), 63.4 (C-6), 47.2 ($N\underline{C}H_2CH_2$), 43.5 ($N\underline{C}H_2$), 28.3 ($NCH_2\underline{C}H_2$); **HR-ESI-QTof** *m*/*z* $[M+H]^+$ calcd. for $C_{45}H_{38}N_3O_{10}$ 780.2552; found 780.2538.

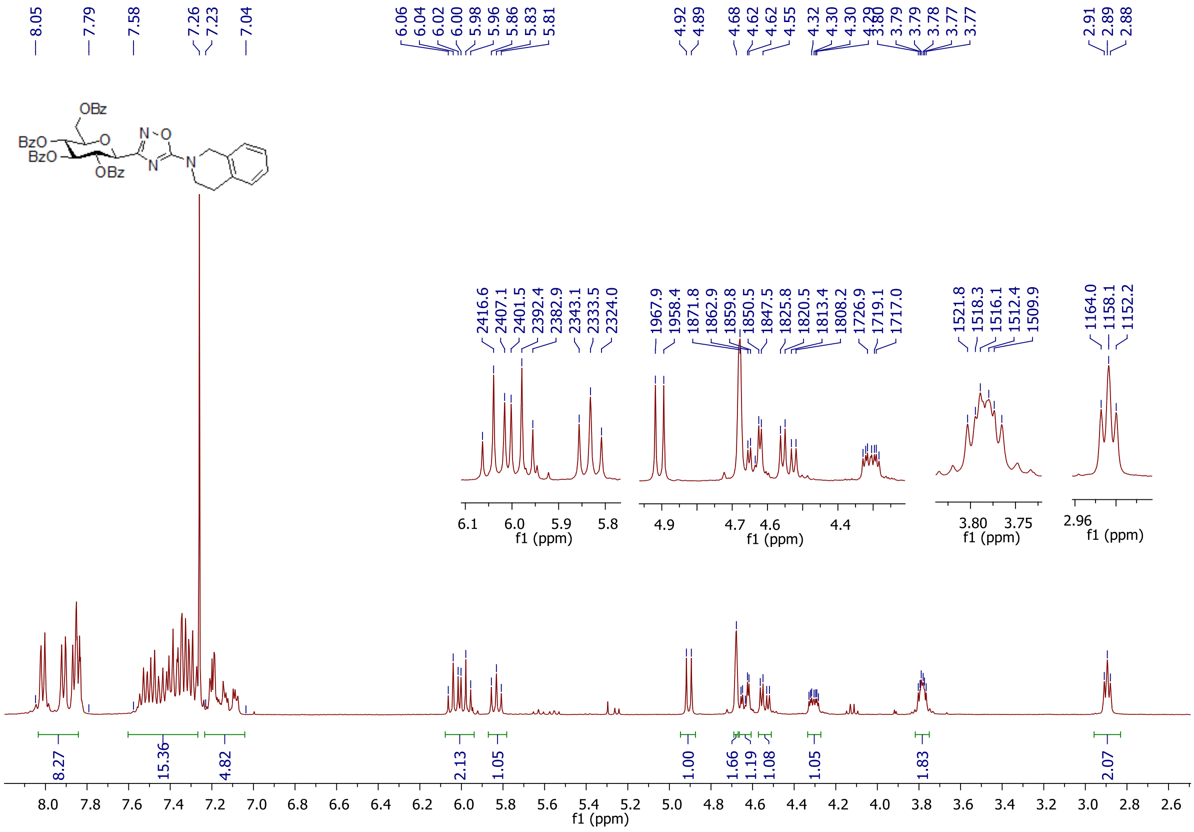
8.05
7.79
7.58
7.26
7.23
7.04
6.06
6.04
6.02
6.00
5.98
5.96
5.86
5.83
5.81
4.92
4.89
4.68
4.62
4.62
4.55
4.32
4.30
4.30
4.29
3.80
3.79
3.79
3.78
3.77
3.77
2.91
2.89
2.88
OBz
BzO
BzO
O
OBz
N-O
N
N
2416.6
2407.1
2401.5
2392.4
2382.9
2343.1
2333.5
2324.0
1967.9
1958.4
1871.8
1862.9
1859.8
1850.5
1847.5
1825.8
1820.5
1813.4
1808.2
1726.9
1719.1
1717.0
1521.8
1518.3
1516.1
1512.4
1509.9
1164.0
1158.1
1152.2
8.27
15.36
4.82
2.13
1.05
1.00
1.66
1.19
1.08
1.05
1.83
2.07
f1 (ppm)

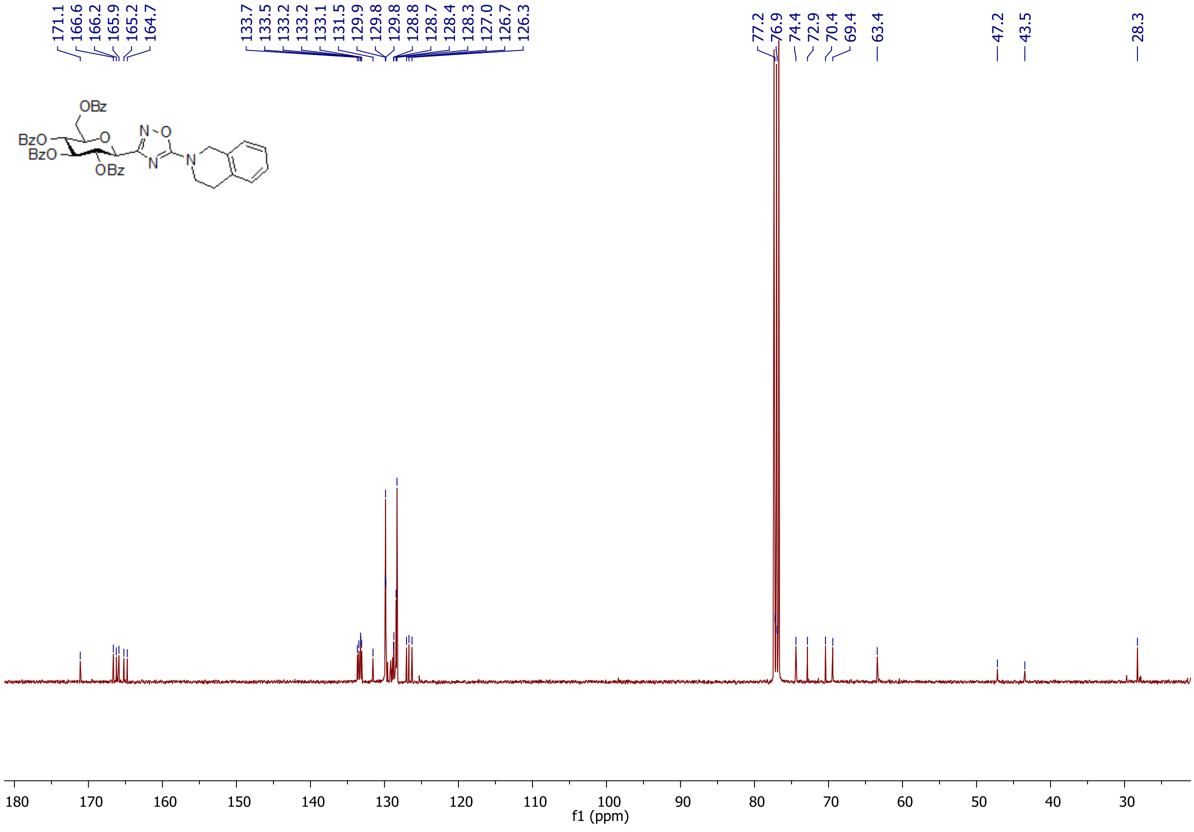
171.1
166.6
166.2
165.9
165.2
164.7
133.7
133.5
133.2
133.2
133.1
131.5
129.9
129.8
129.8
128.8
128.7
128.4
128.3
127.0
126.7
126.3
77.2
76.9
74.4
72.9
70.4
69.4
63.4
47.2
43.5
28.3
OBz
BzO
BzO
O
OBz
N-O
N
N
f1 (ppm)

3-(2,3,4,6-Tetra-*O*-benzoyl-β-D-glucopyranosyl)-5-(morpholin-4-yl)-1,2,4-oxadiazole (4d) Obtained as white foam (65 mg, 0.08 mmol, 29%) following general procedure B: 4-[chloro(morpholino)methylene]morpholin-4-ium chloride (137 mg, 0.63 mmol), 3-(2,3,4,6-tetra-*O*-benzoyl-β-D-glucopyranosyl)-formamidoxime **3** (200 mg, 0.31 mmol), triethylamine (175 µL, 1.25 mmol) and then purified by silica gel column chromatography (EtOAc/PE, 35/65 to 40/60, v/v).

R_f = 0.68 (EtOAc/PE, 40/60, v/v); $[\alpha]_D^{20}$ = -17 (c 1.1, CH_2Cl_2); **^{1}H NMR ($CDCl_3$, 400 MHz)** δ 8.02-7.83 (m, 8H, H-arom), 7.53-7.29 (m, 12H, H-arom), 5.99-5.94 (m, 2H, H-3 and H-2), 5.84-5.78 (m, 1H, H-4), 4.90-4.87 (m, 1H, H-1), 4.60 (dd, 1H, *J* = 12.4 Hz, *J* = 3.0 Hz, H-6a), 4.54(dd, 1H, *J* = 12.4 Hz, *J* = 5.2 Hz, H-6b), 4.33-4.28 (m, 1H, H-5), 3.69 (t, 4H, *J* = 4.7 Hz , NC$\underline{H}_2$), 3.54 (t, 4H, *J* =4.7 Hz, C$\underline{H}_2$O); **^{13}C NMR ($CDCl_3$, 100 MHz)** δ 171.2 (C-5oxa), 166.7 (C-3oxa), 166.2 (C=O), 165.9 (C=O), 165.2, (C=O), 164.7 (C=O), 133.5 (C^{IV}-arom), 133.21 (C^{IV}-arom), 133.17 (C^{IV}-arom), 133.1 (C^{IV}-arom), 129.9-128.3 (m, CH-arom), 76.9 (C-5), 74.4 (C-3), 72.8 (C-1), 70.5 (C-2), 69.4 (C-4), 65.9 (N$\underline{C}H_2$), 63.4 (C-6), 45.8 ($\underline{C}H_2$O); **HR-ESI-QTof** *m/z* $[M+H]^+$ calcd. for $C_{40}H_{36}N_3O_{11}$ 734.2344; found 734.2325.

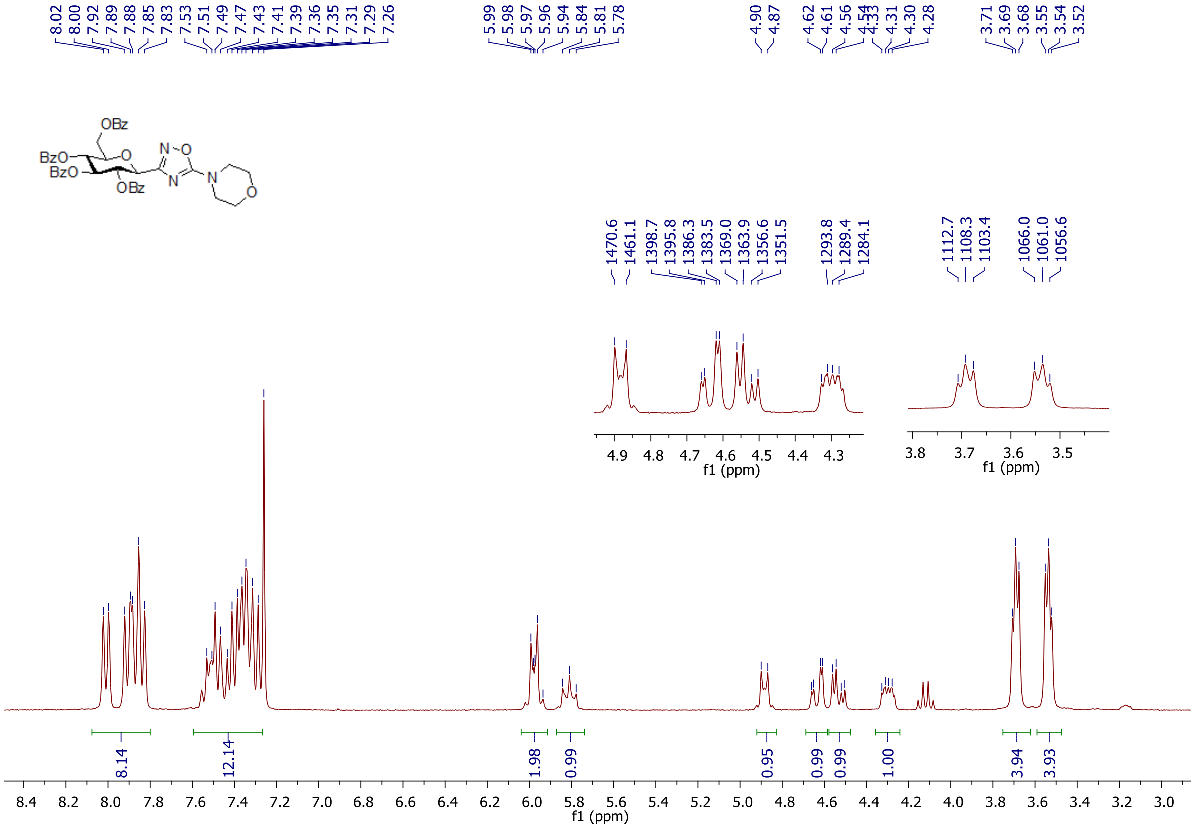

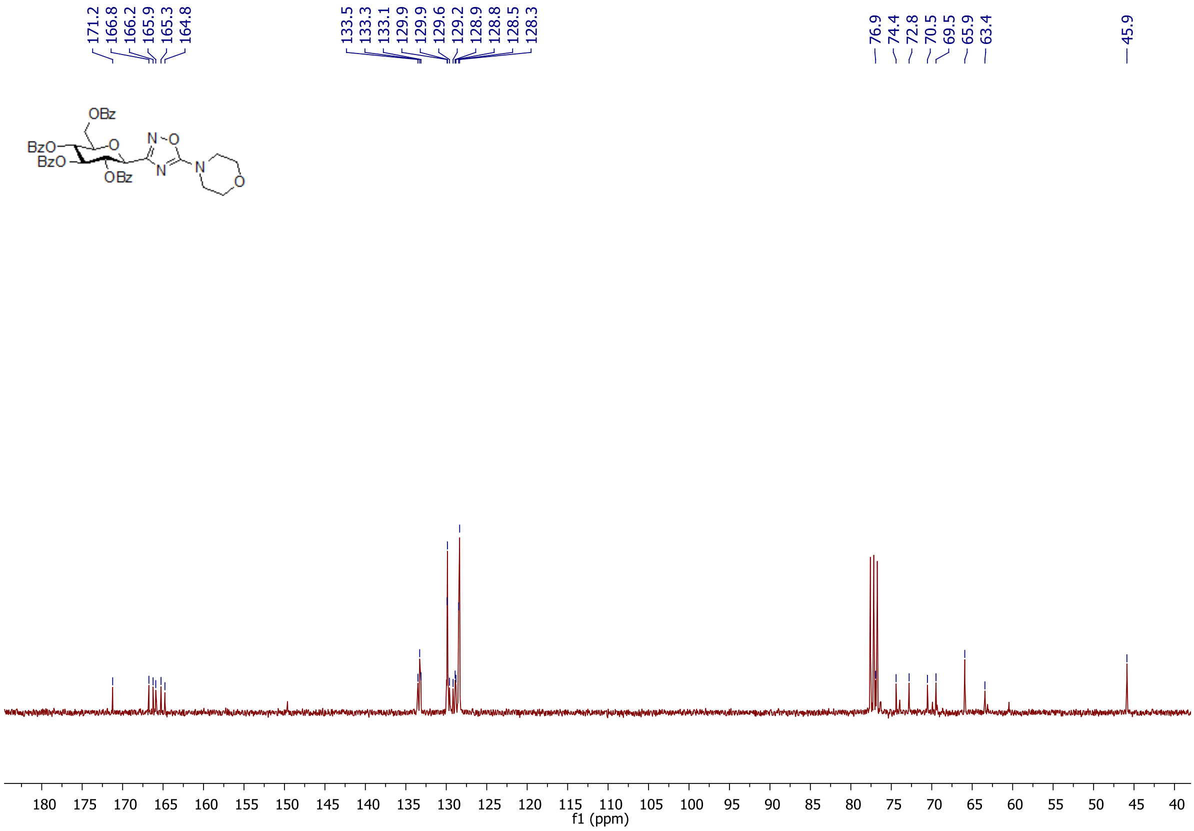

3-(2,3,4,6-Tetra-*O*-benzoyl-β-D-glucopyranosyl)-5-(piperidin-1-yl)-1,2,4-oxadiazole (4e)

Obtained as colorless oil (111 mg, 0.15 mmol, 25%) following general procedure B: *1*-[chloro(piperidino)methylene)piperidin-1-ium chloride (320 mg, 1.27 mmol), 3-(2,3,4,6-tetra-*O*-benzoyl-β-D-glucopyranosyl)-formamidoxime **3** (406 mg, 0.63 mmol), triethylamine (350 µL, 2.5 mmol) and purified by silica gel column chromatography (EtOAc/PE, 35/65 to 40/60, v/v).

R_f = 0.65 (EtOAc/PE, 1/1, v/v); $[\alpha]_D^{20}$ = -8 (c 1.1, CH_2Cl_2); **^{1}H NMR ($CDCl_3$, 400 MHz)** δ 7.96-7.77 (m, 8H, H-arom), 7.47-7.17 (m, 12H, H-arom), 5.99-5.90 (m, 2H, H-3 and H-2), 5.77 (t, 1H,*J* = 9.6 Hz, H-4), 4.84 (d, 1H, *J* = 6.4 Hz, H-1), 4.57(dd, 1H,= 12.3 Hz, *J* = 3.0 Hz, H-6b), 4.50 (dd, 1H, *J* = 12.6 Hz, *J* = 5.2 Hz, H-6a), 4.27-4.24 (m,1H, H-5), 3.43-3.41(m, 4H, NC$\underline{H}_2$), 1.51-1.46 (m, 6H, $CH_2CH_2CH_2$); **^{13}C NMR ($CDCl_3$, 100 MHz)** δ 171.3 (C-5oxa), 166.6 (C-3oxa), 166.2 (C=O), 165.9 (C=O), 165.2, (C=O), 164.7 (C=O), 133.4 (C^{IV}-arom), 133.21 (C^{IV}-arom), 133.18 (C^{IV}-arom), 133.1 (C^{IV}-arom),129.9-128.3 (m, CH-arom), 76.9 (C-5), 74.5 (C-3), 72.9 (C-1), 70.5 (C-2), 69.5 (C-4), 63.4 (C-6), 46.9 (N$\underline{C}H_2$), 25.0 ($NCH_2\underline{C}H_2$), 23.6 ($NCH_2CH_2\underline{C}H_2$); **HR-ESI-QTof** *m*/*z* $[M+H]^+$ calcd. for $C_{41}H_{38}N_3O_{10}$ 732.2552; found 732.2519.

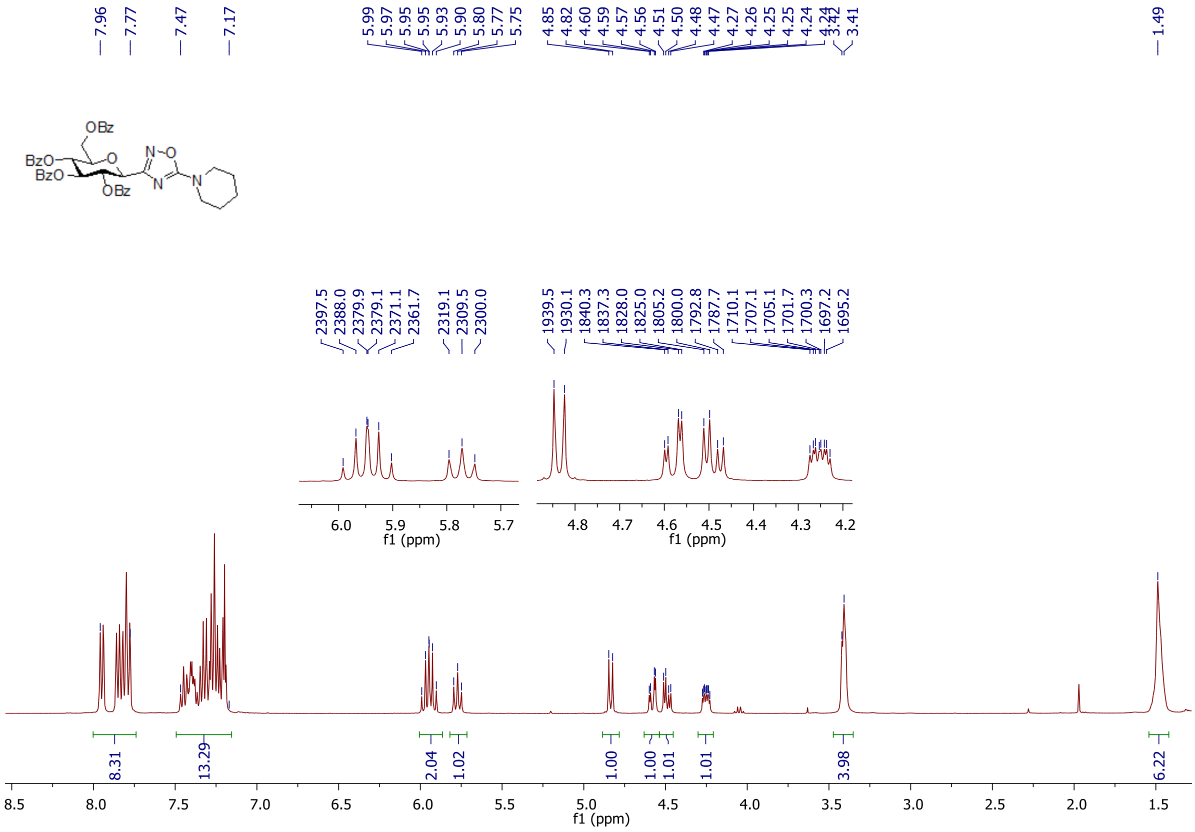

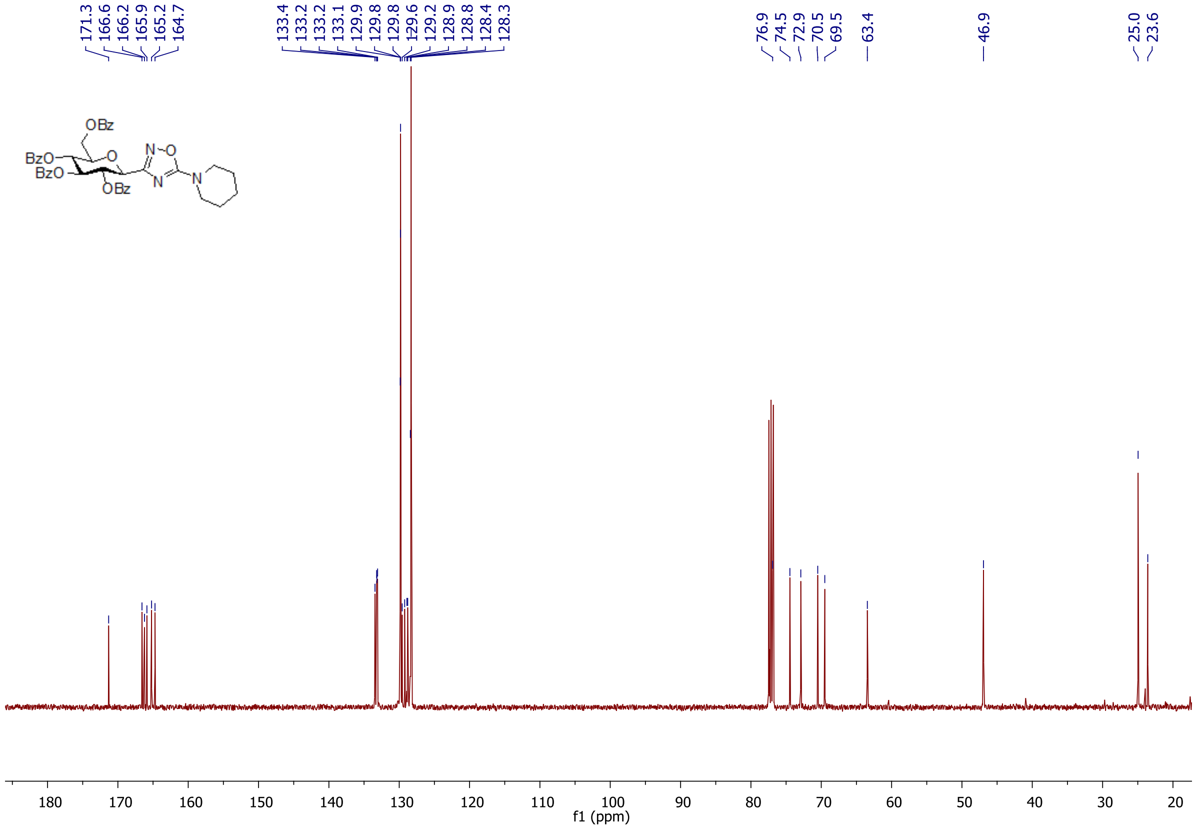

3-(β-D-Glucopyranosyl)-5-(*N*,*N*-dimethylamino)-1,2,4-oxadiazole (5a)

Obtained as white foam (84 mg, 0.30 mmol, 91%) following general procedure C: 3-(2,3,4,6-Tetra-*O*-benzoyl-β-D-glucopyranosyl)-5-(*N*,*N*-dimethylamino)-1,2,4-oxadiazole (**4a**, 230 mg, 0.33 mmol) and purified by silica gel column chromatography (EtOAc to CH_2Cl_2/MeOH, 80/20, v/v).

R_f = 0.3 (PE/AcOEt, 60/40, v/v); $[\alpha]_D^{20}$ = +3 (c 0.7, MeOH); **^{1}H NMR (CD_3OD, 400 MHz)** δ 4.18 (d, 1H, *J* = 9.7 Hz, H-1), 3.86 (dd, 1H, *J* = 12.2 Hz, *J* = 2.0 Hz, H-6a), 3.72-3.65 (m, 2H, H-2 and H-6b), 3.47-3.41 (m, 3H, H-5, H-3, H-4), 3.17 (s, 6H, NCH_3); **^{13}C NMR (CD_3OD, 100 MHz)** δ 173.0 (C-5oxa), 169.5 (C-3oxa), 82.3 (C-5), 79.1 (C-3), 75.2 (C-1), 73.2 (C-2), 71.0 (C-4), 62.7 (C-6), 38.3 (NCH_3); **HR-ESI-QTof** *m/z* $[M+Na]^+$ calcd. for $C_{17}H_{21}N_3NaO_6$ 386.1323; found 386.1306.

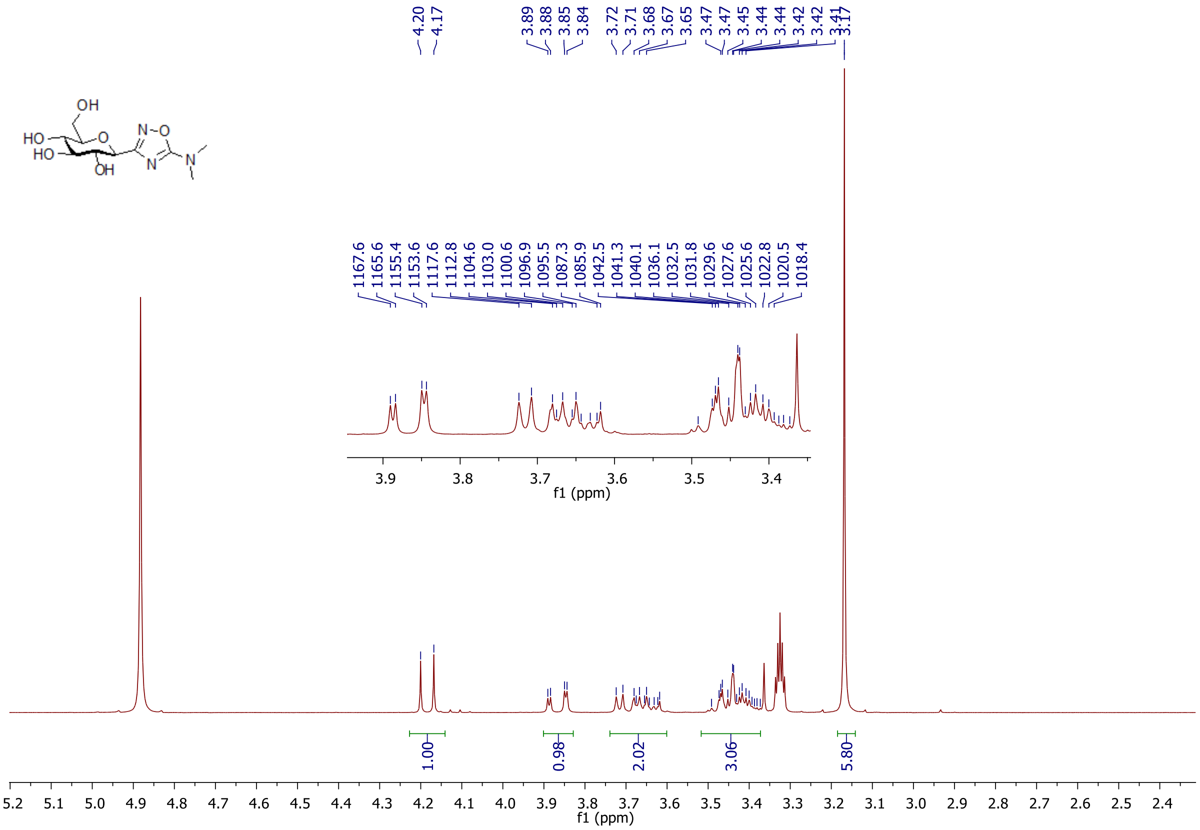

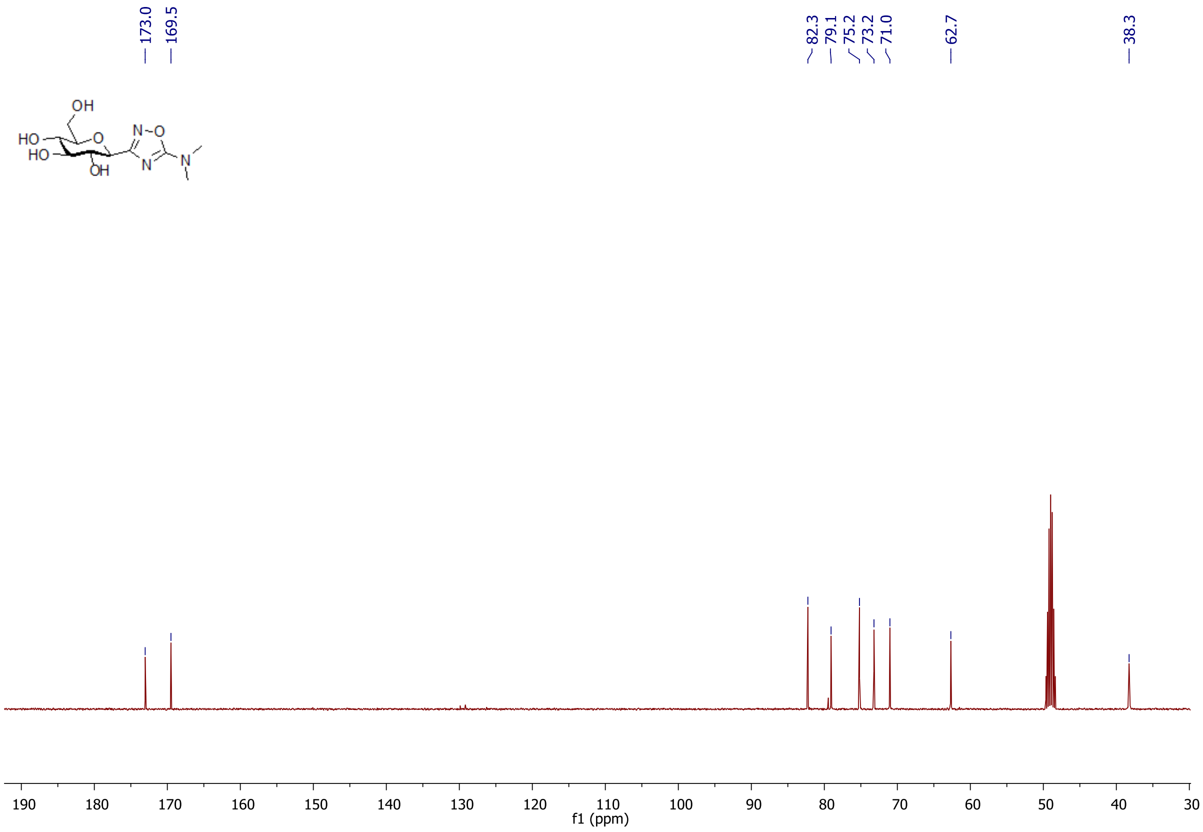

3-(β-D-Glucopyranosyl)-5-(*N*-methyl-*N*-benzylamino)-1,2,4-oxadiazole (5b)

Obtained as colorless oil (8 mg, 0.022 mmol, 62%) following general procedure C: 3-(2,3,4,6-Tetra-*O*-benzoyl-β-D-glucopyranosyl)-5-(*N*-methyl-*N*-benzylamino)-1,2,4-oxadiazole (**4b**, 29 mg, 0.037 mmol) and purified by thick-layer chromatography (CH_2Cl_2/MeOH, 80/20, v/v). R_f = 0.8 (CH_2Cl_2/MeOH, 80/20, v/v); $[\alpha]_D^{25}$ = +6 (c 0.3, MeOH); **^{1}H NMR (CD_3OD, 300 MHz)** δ 7.38-7.32 (m, 5H, H-arom), 4.72 (s, 2H, C<u>H</u>$_2$Ph), 4.22 (d, 1H, *J* =9.7 Hz, H-1), 3.90 (dd, 1H, *J* = 12.3 Hz, *J* = 1.7 Hz, H-6a), 3.73-3.69 (m, 2H, H-2 and H-6b), 3.48-3.43 (m, 3H, H-5, H-3, H-4), 3.10 (s, 3H, NCH_3); **^{13}C NMR (CD_3OD, 100 MHz)** δ 173.3 (C-5oxa), 169.7 (C-3oxa), 137.1 (C^{IV}-arom), 129.9 (CH-arom), 129.1 (CH-arom), 128.8 (CH-arom), 82.4 (C-5), 79.2 (C-3), 75.4, (C-1), 73.3 (C-2), 71.2 (C-4), 62.7 (C-6), 55.3 (<u>C</u>H$_2$Ph), 35.9 (NCH_3); **HR-ESI-QTof** *m*/*z* $[M+Na]^+$ calcd. for $C_{13}H_{21}N_3NaO_6$ 374.1323; found 374.1313.

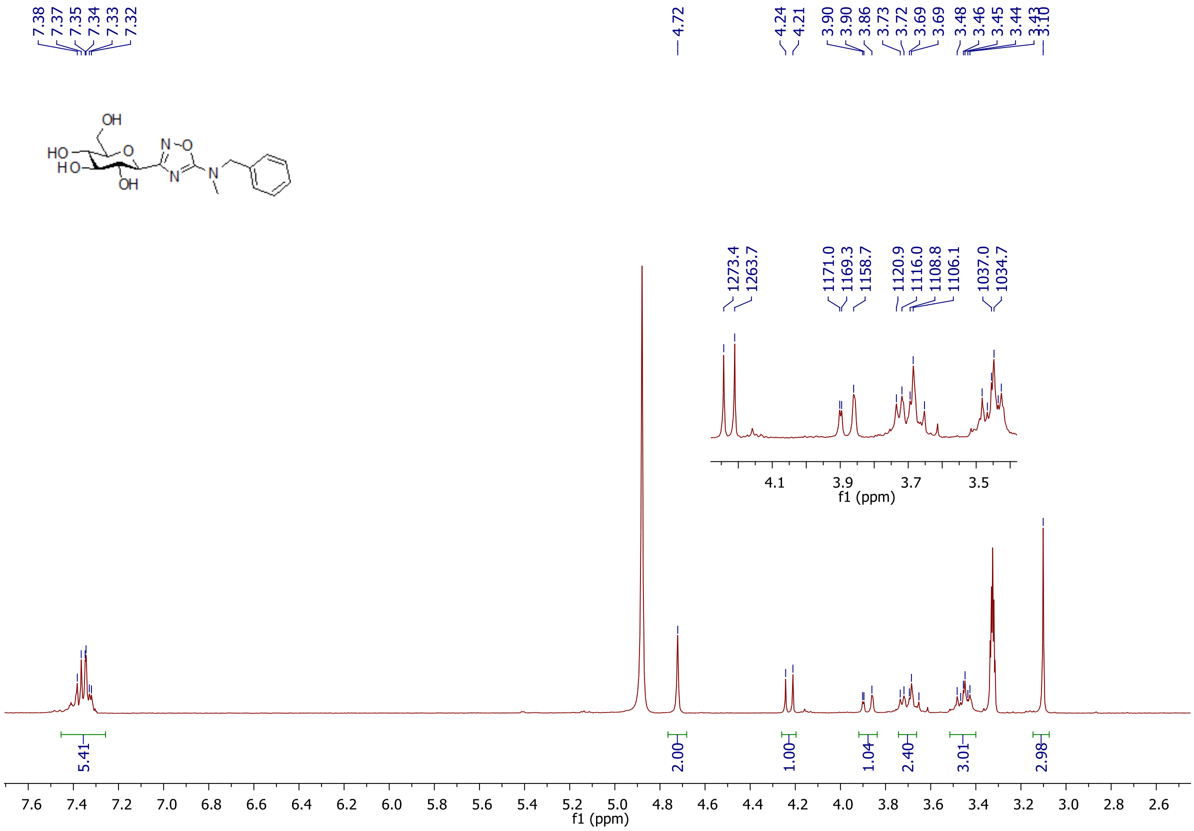
7.38 7.37 7.35 7.34 7.33 7.32
4.72
4.24 4.21
3.90 3.90 3.86 3.73 3.72 3.69 3.69 3.48 3.46 3.45 3.44 3.43 3.10
1273.4 1263.7
1171.0 1169.3 1158.7
1120.9 1116.0 1108.8 1106.1
1037.0 1034.7
f1 (ppm)
5.41 2.00 1.00 1.04 2.40 3.01 2.98
f1 (ppm)

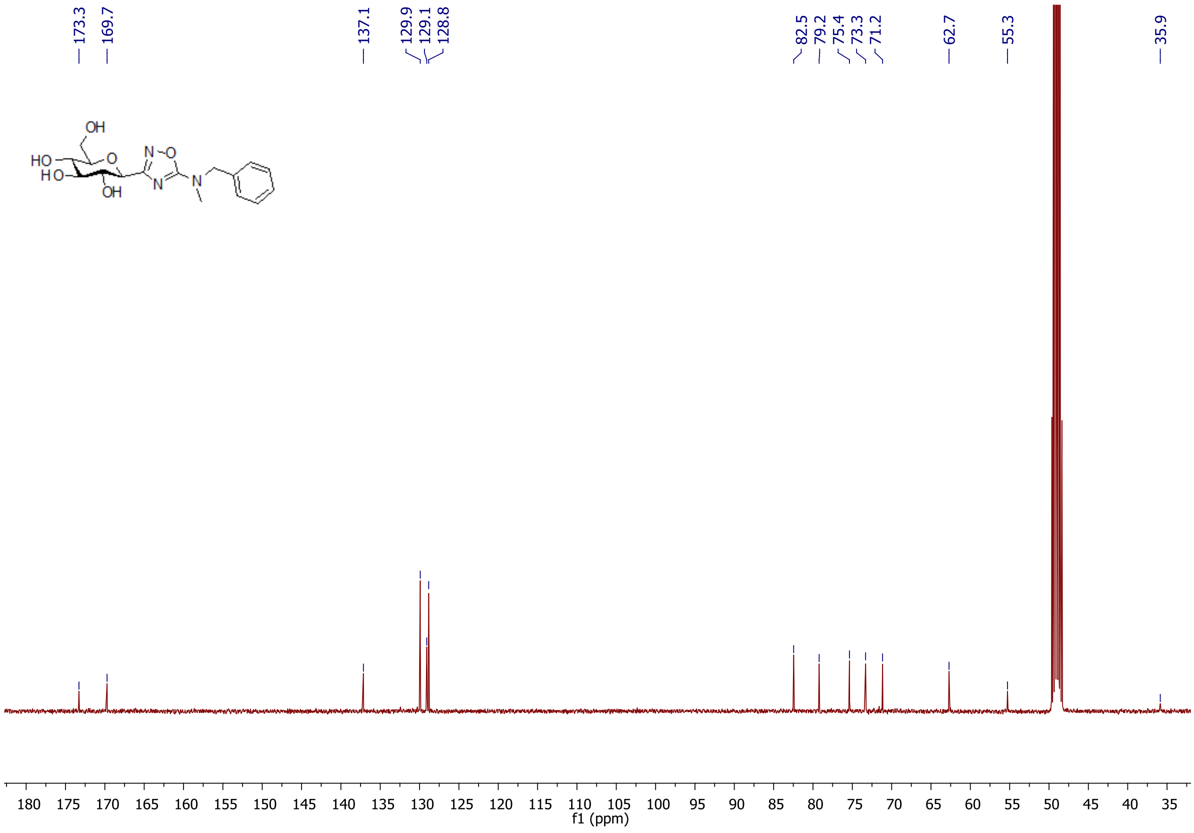
173.3 169.7
137.1
129.9 129.1 128.8
82.5 79.2 75.4 73.3 71.2
62.7
55.3
35.9
f1 (ppm)

3-(β-D-Glucopyranosyl)-5-(1,2,3,4-tetrahydroisoquinolin-2-yl)-1,2,4-oxadiazole (5c)

Obtained as white foam (9 mg, 0.024 mmol, 37%) following general procedure C: 3-(2,3,4,6-Tetra-*O*-benzoyl-β-D-glucopyranosyl)-5-(1,2,3,4-tetrahydroisoquinolin-2-yl)-1,2,4-oxadiazole (**4c**, 53 mg, 0.067 mmol) and purified by silica gel column chromatography (EtOAc to CH_2Cl_2/MeOH, 80/20, v/v).

R_f = 0.3 (PE/AcOEt, 60/40, v/v); $[\alpha]_D^{20}$ = +4 (c 0.7, MeOH); **^{1}H NMR (CD_3OD, 400 MHz)** δ 7.21 (m, 4H, H-arom), 4.77 (s, 2H, NCH_2), 4.20 (d, 1H, *J* = 9.7 Hz, H-1), 3.88-3.85 (m, 3H, $NC\underline{H}_2CH_2$ and H-6a), 3.72-3.67 (m, 2H, H-2 and H-6b), 3.46-3.35 (m, 3H, H-3, H-4 and H-5), 2.99 (t, 2H, *J* = 6.0 Hz, $NCH_2C\underline{H}_2$); **^{13}C NMR (CD_3OD, 100 MHz)**δ 172.4 (C-5oxa), 169.6 (C-3oxa), 135.2 (C^{IV}-arom), 133.1 (C^{IV}-arom), 129.8 (CH-arom), 128.2 (CH-arom), 127.7 (CH-arom), 127.3 (CH-arom), 82.5 (C-5), 79.2 (C-3), 75.4 (C-1), 73.3 (C-2), 71.2 (C-4), 62.8 (C-6), 48.4 (NCH_2), 44.9 ($N\underline{C}H_2CH_2$), 29.1 ($NCH_2\underline{C}H_2$); **HR-ESI-QTof** *m*/*z* $[M+Na]^+$ calcd. for $C_{17}H_{21}N_3NaO_6$ 386.1323; found 386.1306.

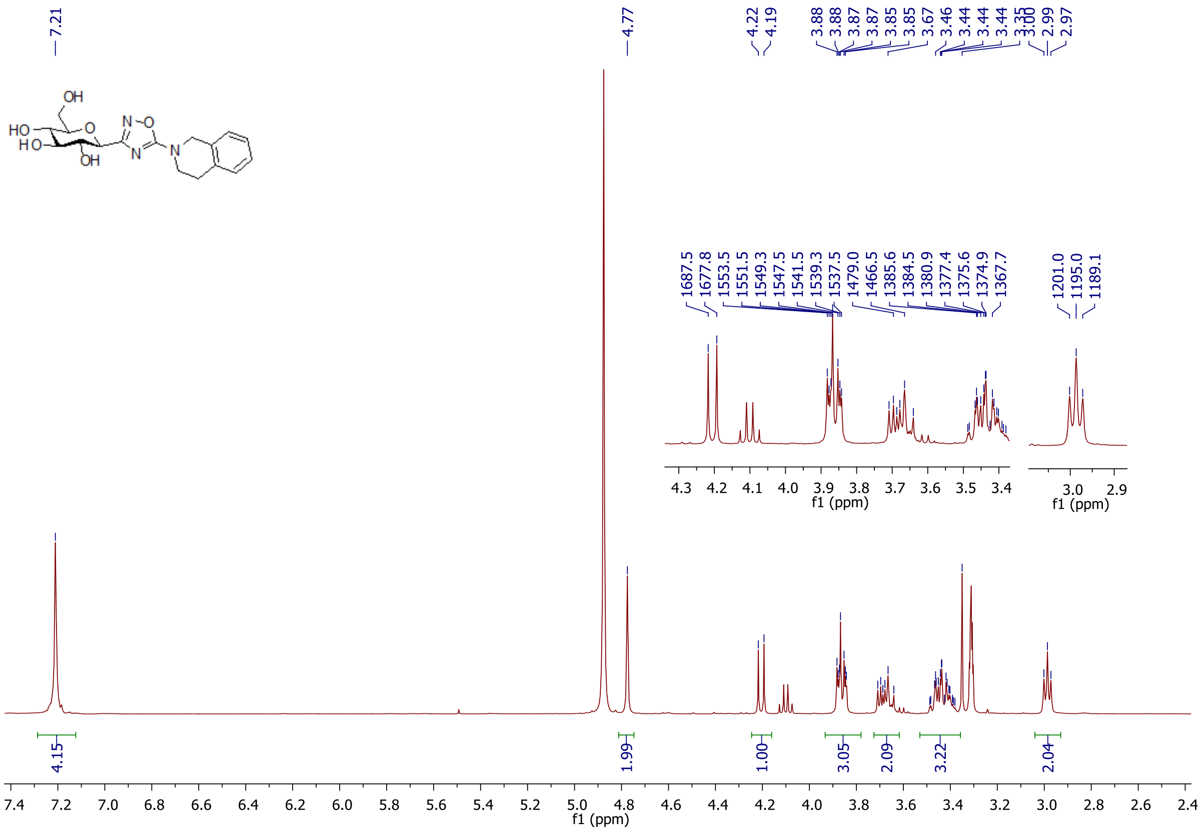

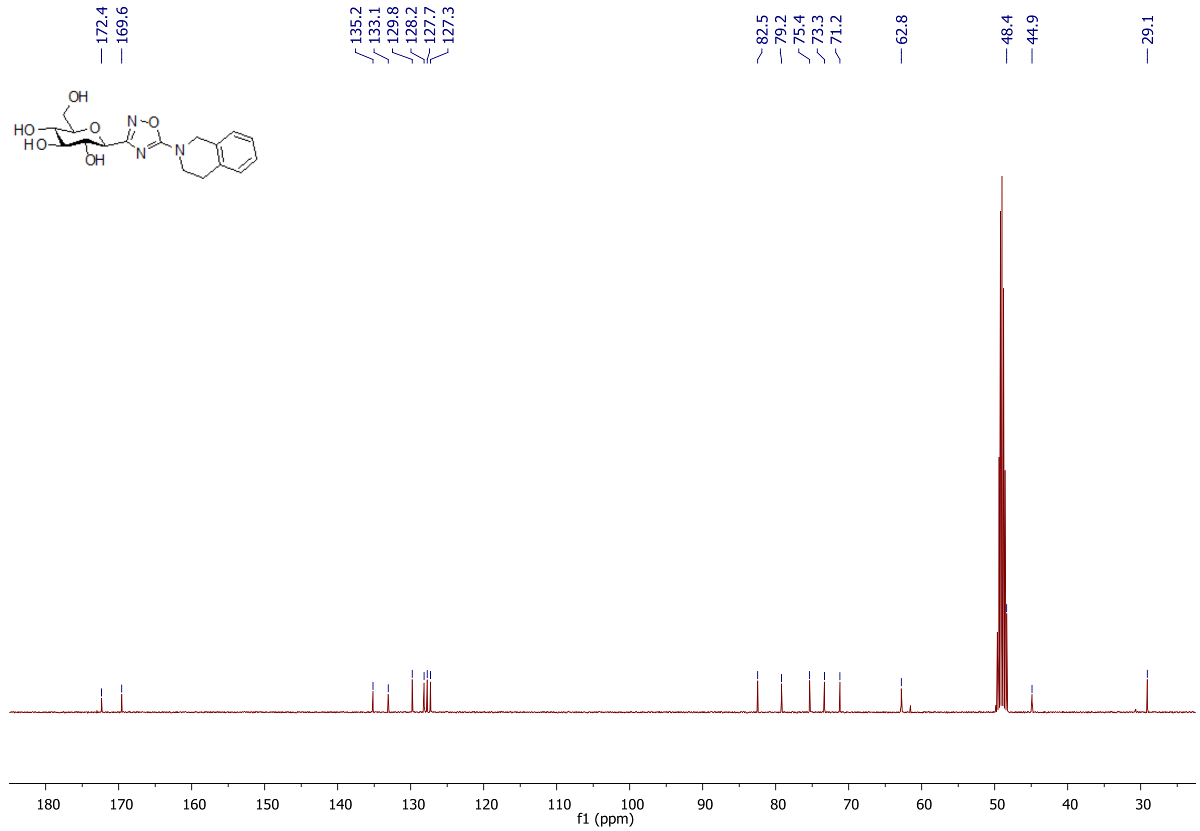

3-(β-D-Glucopyranosyl)-5-(morpholin-4-yl)-1,2,4-oxadiazole (5d)

Obtained as white foam (10 mg, 0.03 mmol, 67%) following general procedure C: 3-(2,3,4,6-Tetra-*O*-benzoyl-β-D-glucopyranosyl)-5-(morpholin-4-yl)-1,2,4-oxadiazole (**4d**, 35 mg, 0.05 mmol) and purified by silica gel column chromatography (EtOAc to CH_2Cl_2/MeOH, 80/20, v/v).

R_f = 0.3 (PE/AcOEt, 60/40, v/v); $[\alpha]_D^{20}$ = +9 (c 1.1, MeOH); **^{1}H NMR (CD_3OD, 400 MHz)** δ 4.19 (d, 1H, *J* = 9.7 Hz, H-1), 3.85 (m, 1H, H-6a), 3.78-3.75 (m, 4H, $C\underline{H}_2O$), 3.70-3.66 (m, 1H, H-6b), 3.63-3.61 (m, 4H, NCH_2), 3.48-3.40 (m, 3H, H-3, H-4, H-5);**^{13}C NMR (CD_3OD, 100 MHz)**δ 172.5 (C-5oxa), 169.6 (C-3oxa), 82.5 (C-5), 79.2 (C-3), 75.3 (C-1), 73.3 (C-2), 71.2 (C-4), 67.0 (NCH_2), 62.8 (C-6), 47.1 (CH_2O); **HR-ESI-QTof** *m*/*z* $[M+Na]^+$ calcd. for $C_{12}H_{19}N_3NaO_7$ 340.1115; found 340.1128.

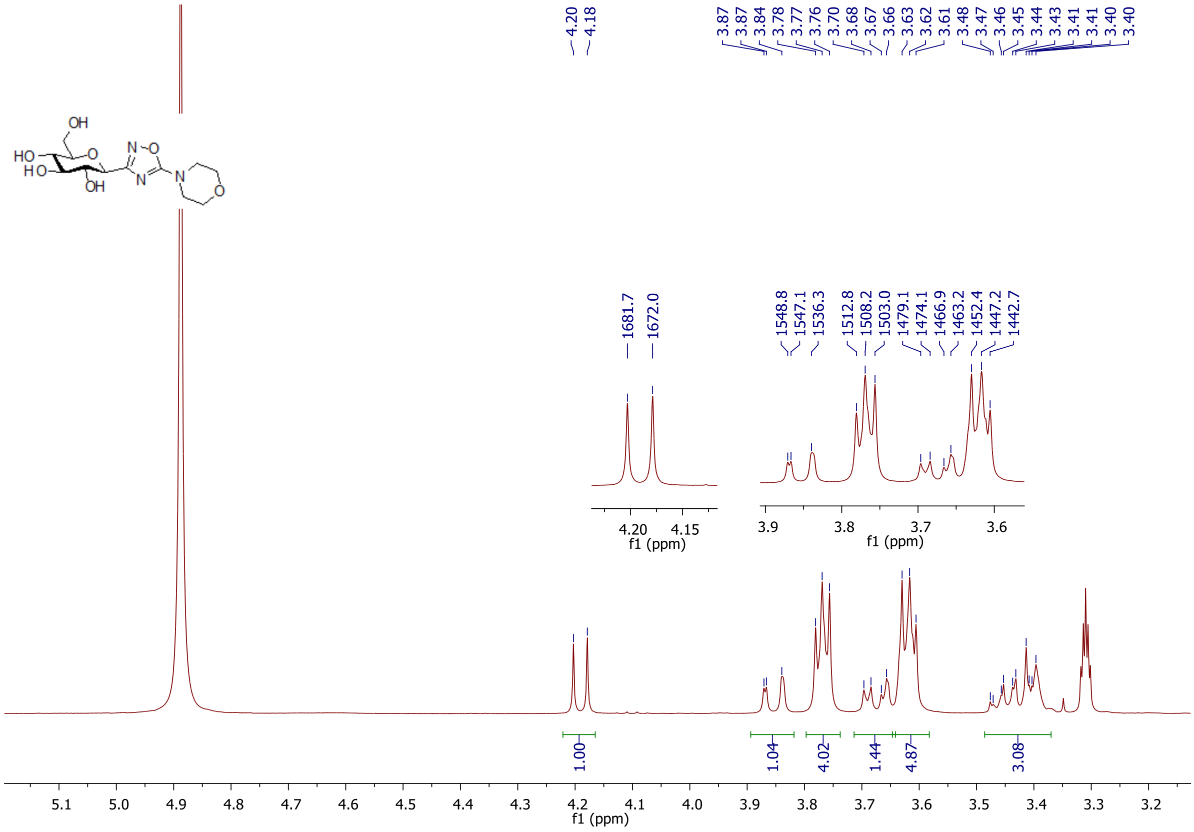

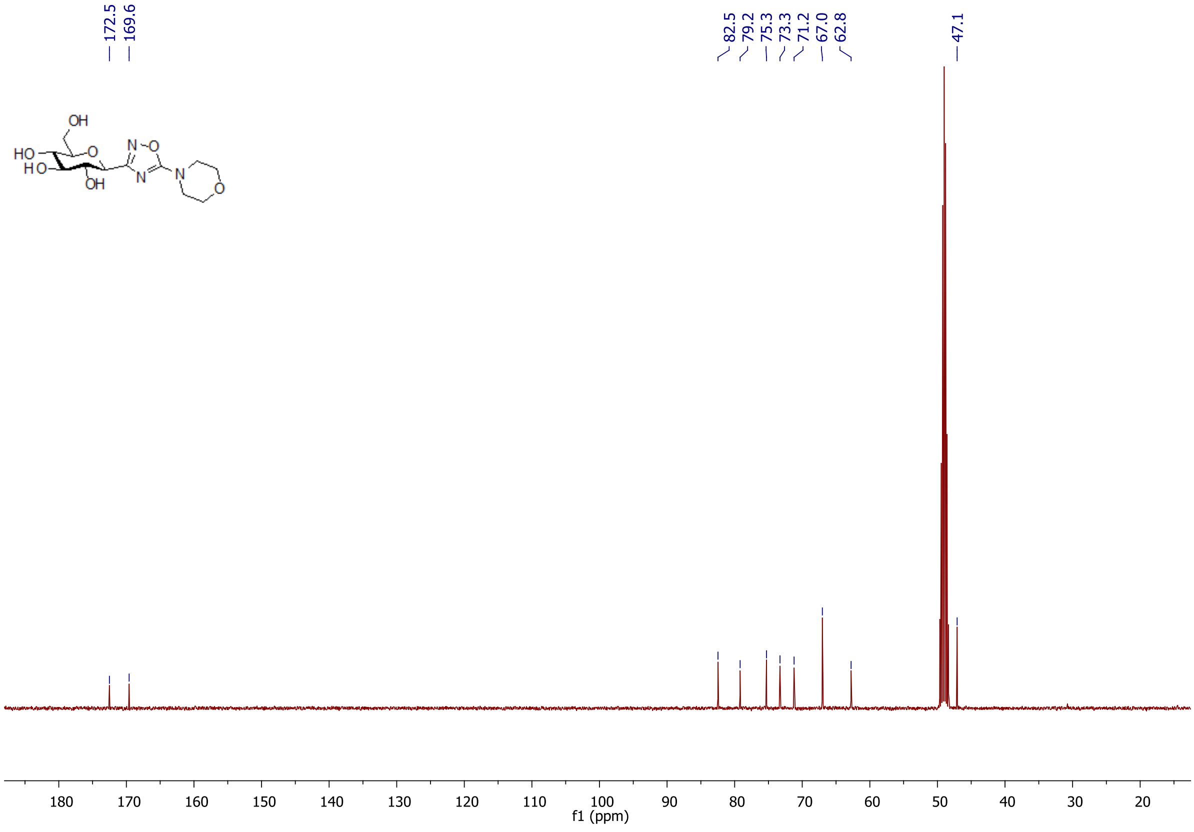

3-(β-D-Glucopyranosyl)-5-(piperidin-1-yl)-1,2,4-oxadiazole (5e)

Obtained as slightly purple oil (7.5 mg, 0.023 mmol, 79%) following general procedure C: 3-(2,3,4,6-Tetra-*O*-benzoyl-β-D-glucopyranosyl)-5-(piperidin-1-yl)-1,2,4-oxadiazole (**4e**, 22 mg, 0.030 mmol) and purified by silica gel column chromatography (EtOAc to CH_2Cl_2/MeOH, 80/20, v/v).

R_f = 0.35 (CH_2Cl_2/MeOH, 80/20, v/v); $[\alpha]_D^{20}$ = +5 (c 0.13, MeOH); **^{1}H NMR (CD_3OD, 300 MHz)** δ 4.17 (d, 1H, *J* =4.4 Hz, H-1), 3.85 (dd, 1H, *J* = 12.0 Hz, *J* = 1.8 Hz, H-6a), 3.63-3.59 (m, 6H, $N(CH_2)_2$, H-2 and H-6b), 3.45-3.39 (m, 3H, H-5, H-3, H-4), 1.69 (s, 6H, $CH_2CH_2CH_2$); **^{13}C NMR (CD_3OD, 100 MHz)**δ 172.4 (C-5oxa), 169.5 (C-3oxa), 82.4 (C-5), 79.2 (C-3), 75.4, (C-1), 73.3 (C-2), 71.2 (C-4), 62.8 (C-6), 48.1 (NCH_2), 26.3 ($NCH_2\underline{C}H_2$), 24.6 ($NCH_2CH_2\underline{C}H_2$); **HR-ESI-QTof** *m/z* $[M+Na]^+$ calcd. for $C_{13}H_{21}N_3NaO_6$ 338.1323; found 338.1325.

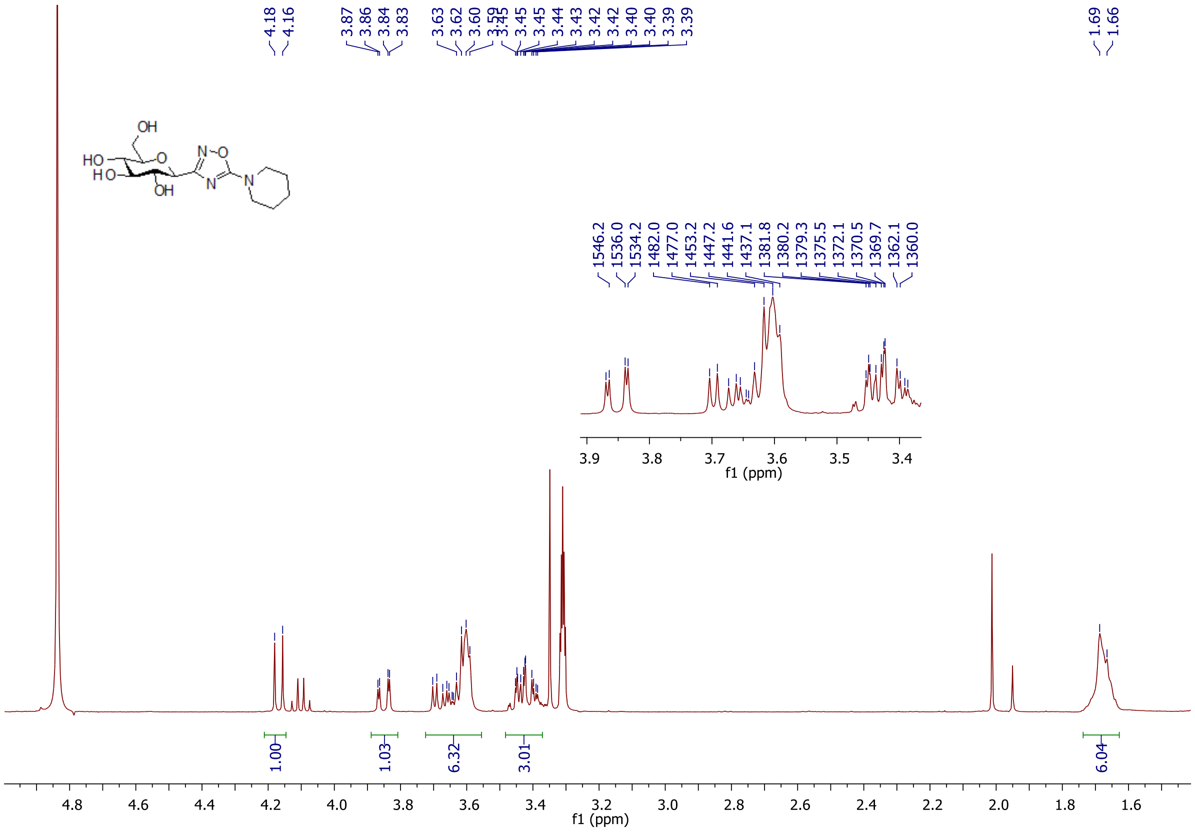

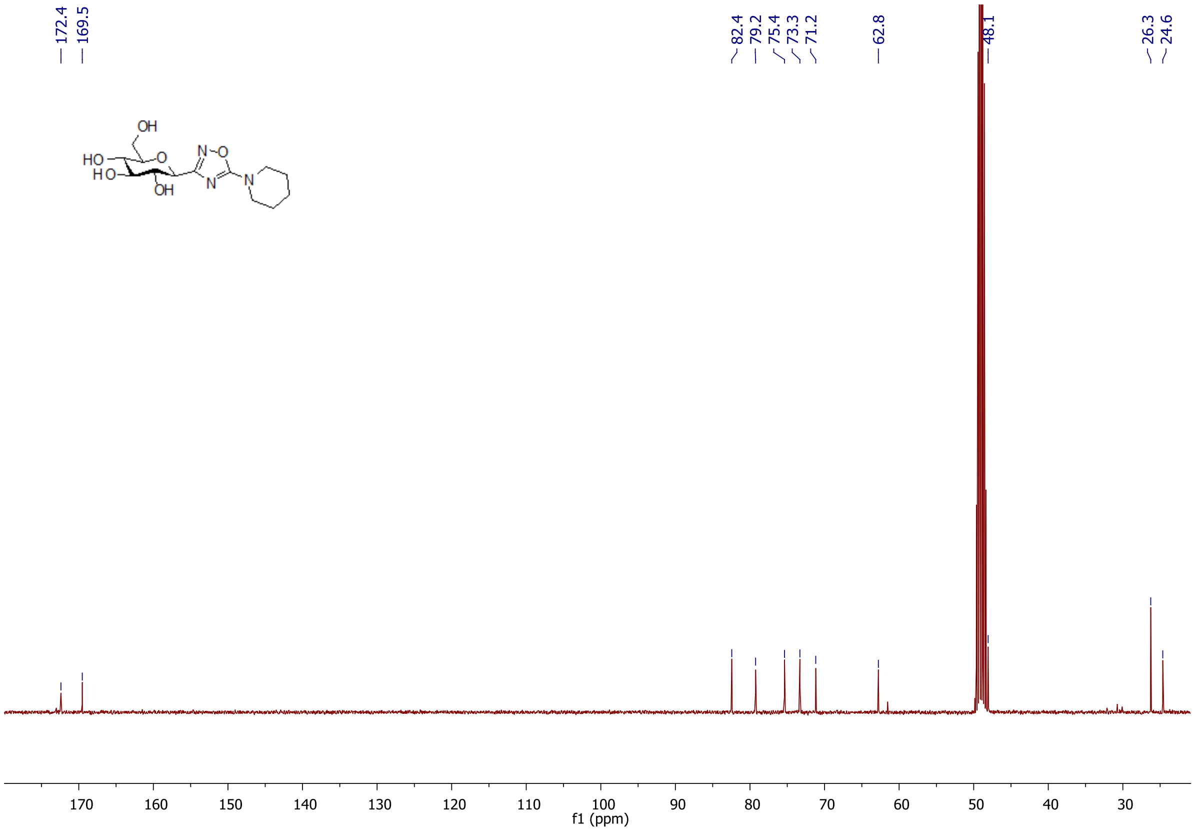

3-(β-D-Glucopyranosyl)-5-isopropylamino-1,2,4-oxadiazole (7a)

Obtained according to the general procedure D as a white powder (46 mg, 0.16 mmol, >95%). R_f = 0.15 (CH_2Cl_2/MeOH 9/1); mp = 206-207°C (CH_2Cl_2/PE); $[\alpha]_D^{20}$ = +150 (c 1.1, MeOH); **^{1}H NMR (CD_3OD, 400 MHz)** δ 4.18 (d, 1H, *J* = 9.7 Hz, H-1), 3.85-3.91 (m, 1H, C<u>H</u>(CH_3)$_2$), 3.86 (dd, 1H, *J* = 12.1, 2.0 Hz, H-6a), 3.69 (dd, 1H, *J* = 12.1, 4.9 Hz, H-6b), 3.65 (m, 1H, H-2), 3.45 (m, 2H, H-3, H-4), 3.39 (ddd, 1H, *J* = 10.0, 5.1, 2.1 Hz, H-5), 1.26 (d, 6H, *J* = 6.5 Hz, CH(C<u>H</u>$_3$)$_2$); **^{13}C NMR (CD_3OD, 100 MHz)** δ 172.4 (C-5oxa), 169.1 (C-3oxa), 82.4 (C-5), 79.3 (C-3), 75.3 (C-1), 73.3 (C-2), 71.1 (C-4), 62.8 (C-6), 47.2 (<u>C</u>H(CH_3)$_2$), 22.9 (CH(<u>C</u>H$_3$)$_2$), 22.8 (CH(<u>C</u>H$_3$)$_2$); **HR-ESI-QTof** *m/z* calcd. for $[M+Na]^+$ $C_{11}H_{19}N_3NaO_6$ 312.1166; found 312.1166.

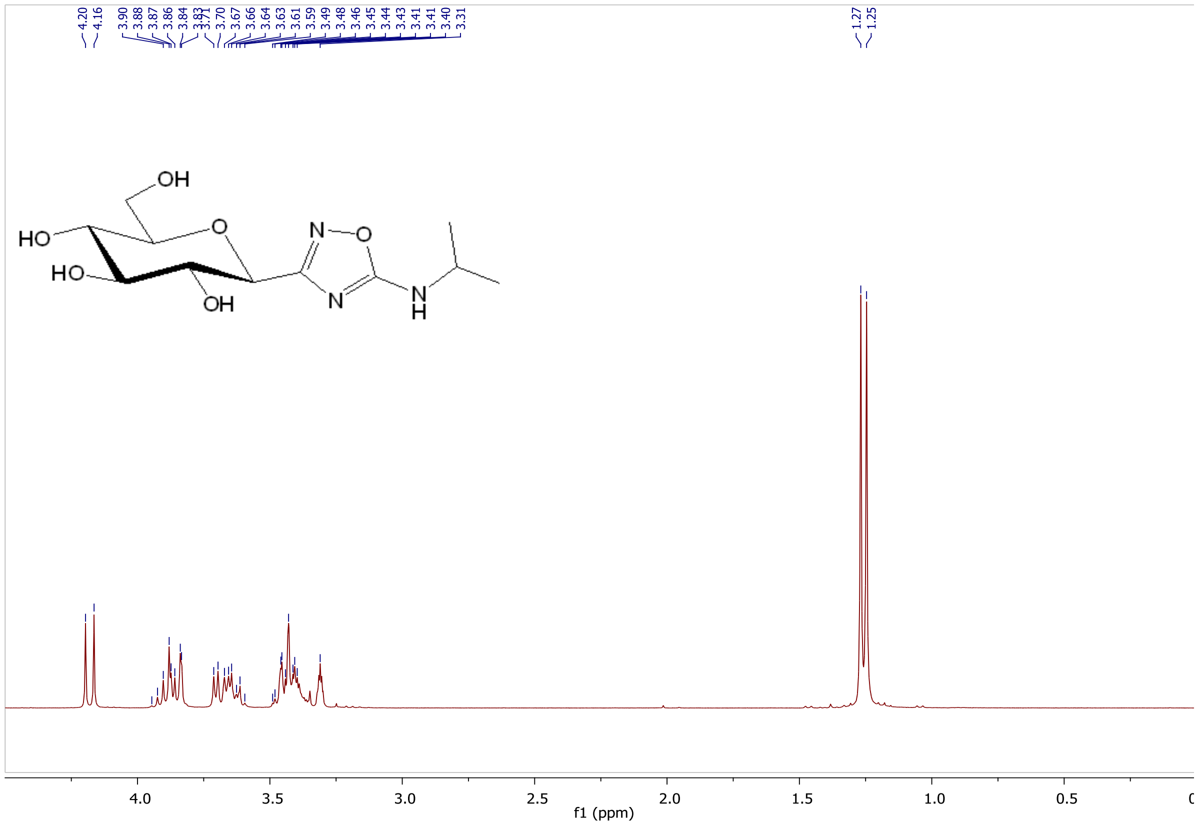
f1 (ppm)

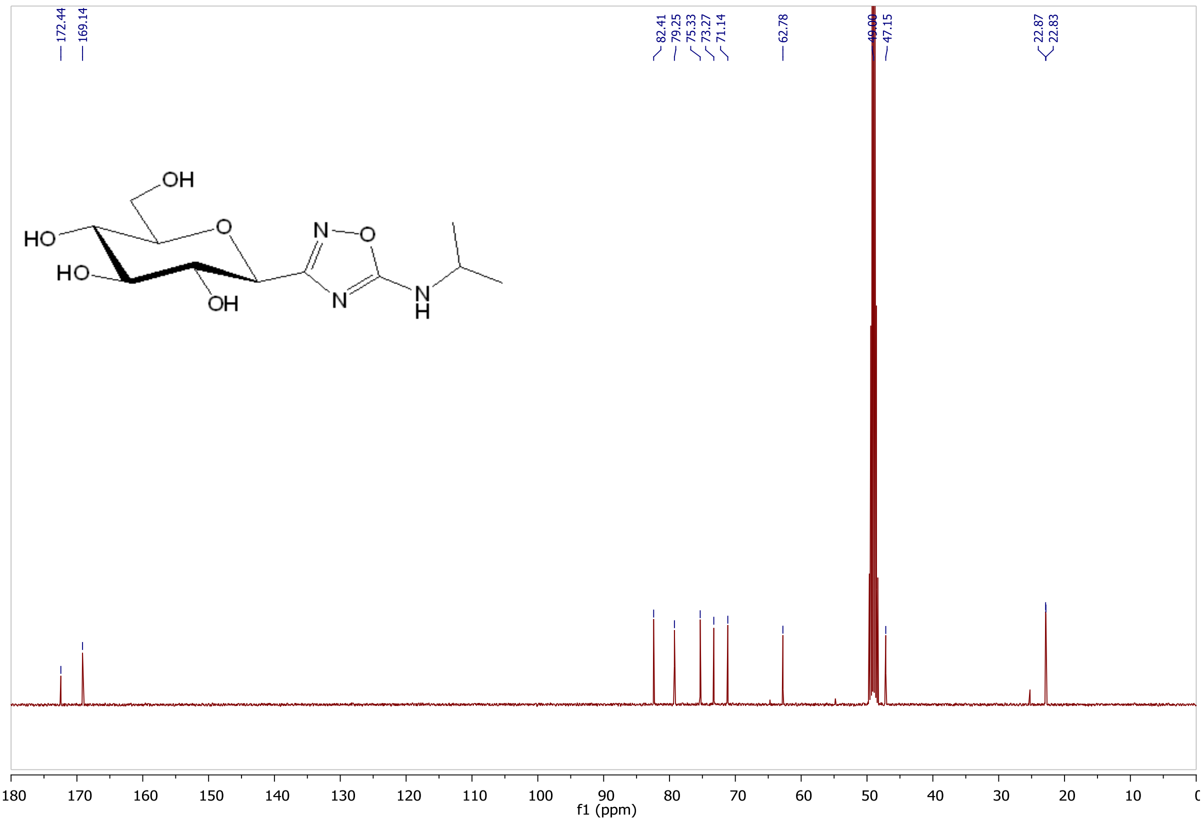
f1 (ppm)

5-(Cyclohexylamino)-3-(β-D-glucopyranosyl)-1,2,4-oxadiazole (7b)

Obtained according to the general procedure D as a white powder (53 mg, 0.16 mmol, >95%). R_f = 0.13 (CH_2Cl_2/MeOH 9/1); mp = 213-214°C (CH_2Cl_2/PE); $[\alpha]_D^{20}$ = +139 (c 1, MeOH); **^{1}H NMR (CD_3OD, 400 MHz)** δ 4.18 (d, 1H, *J* = 9.7 Hz, H-1), 3.86 (dd, 1H, *J* = 12.2, 1.9 Hz, H-6a), 3.69 (dd, 1H, *J* = 12.0, 4.9 Hz, H-6b), 3.64 (dd, 1H, *J* = 9.6, 4.6 Hz, H-2), 3.51-3.57 (m, 1H, H-Cy) 3.36-3.48 (m, 3H, H-3, H-4, H-5), 1.99-2.02 (m, 2H, H-Cy), 1.78-1.81 (m, 2H, H-Cy), 1.64-1.68 (m, 1H, H-Cy), 1.18-1.45 (m, 5H, H-Cy); **^{13}C NMR (CD_3OD, 100 MHz)** δ 172.5 (C-5oxa), 169.1 (C-3oxa), 82.4 (C-5), 79.3 (C-3), 75.3 (C-1), 73.3 (C-2), 71.2 (C-4), 62.8 (C-6), 54.2 (CH, cyclohexyl), 34.0 (CH_2, cyclohexyl), 34.0 (CH_2, cyclohexyl), 26.5 (CH_2, cyclohexyl), 26.0 (CH_2, cyclohexyl); **HR-ESI-QT of** *m/z* calcd. for $[M+H]^+$ $C_{14}H_{24}N_3O_6$ 330.1660; found 330.1660.

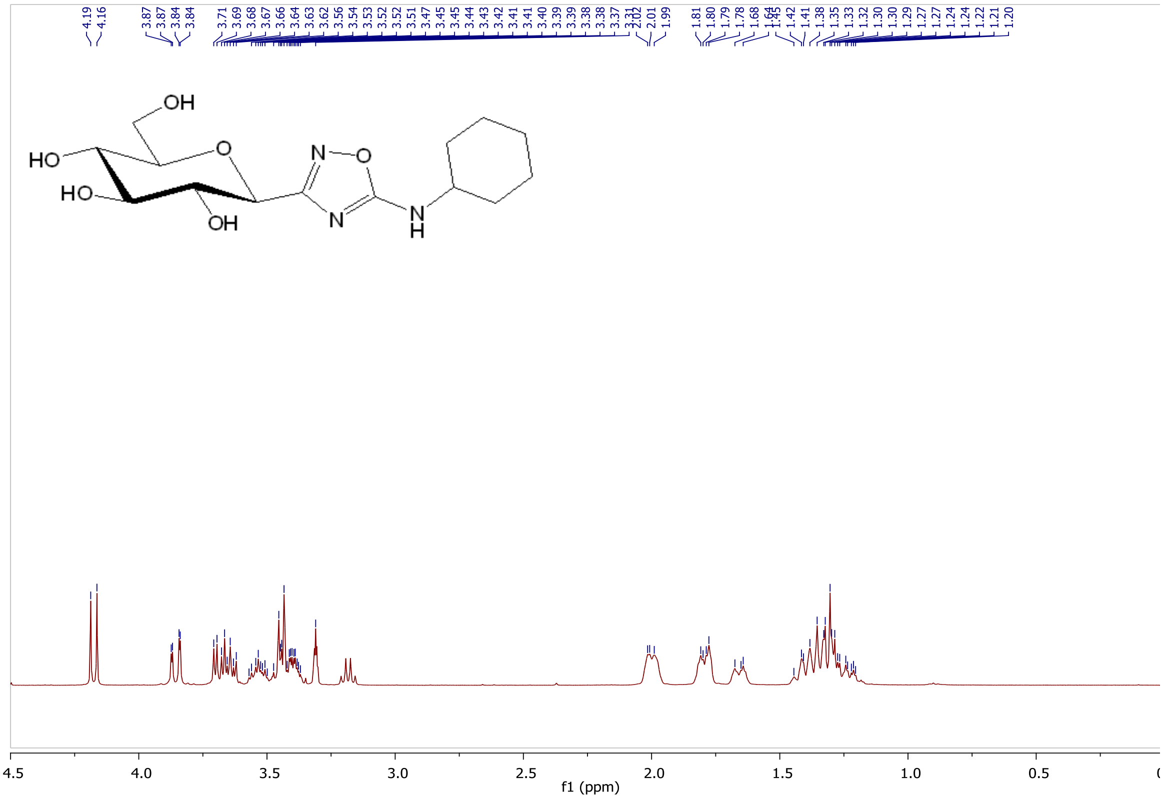

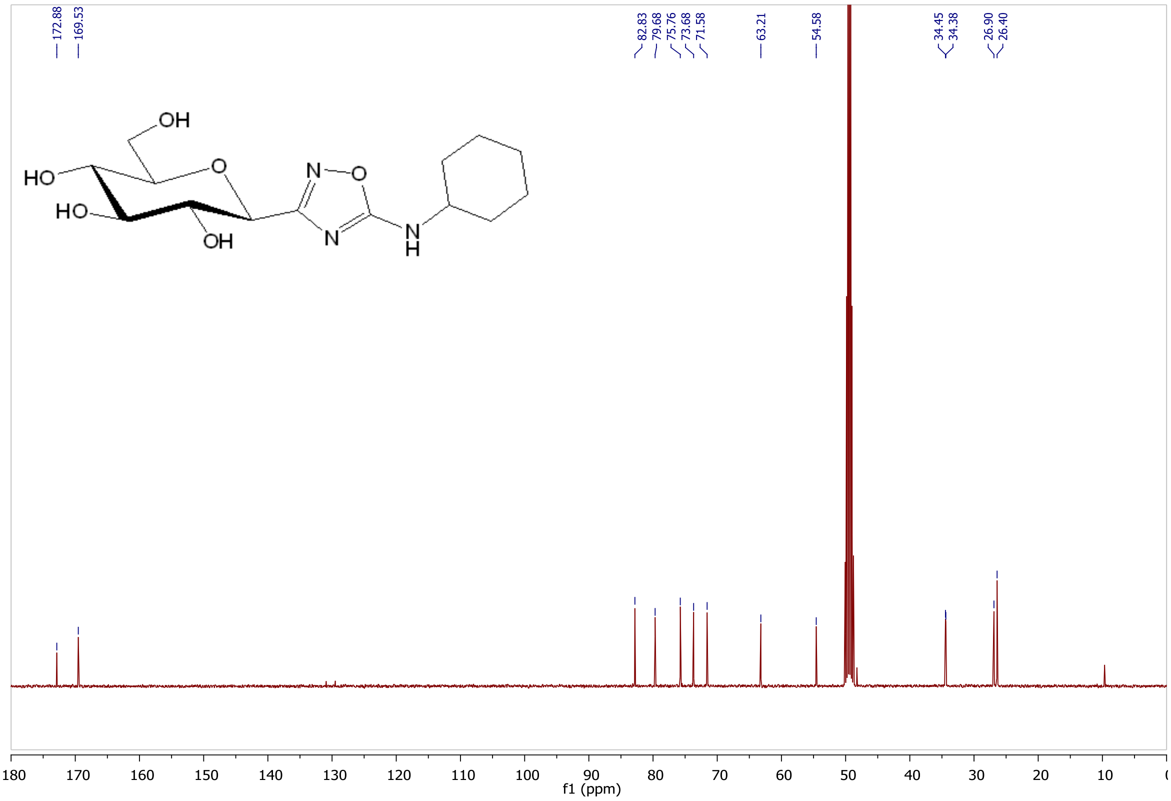

Enzymatic inhibition

Kinetic experiments were performed in the direction of glycogen synthesis as described previously (Oikonomakos, N. G.; Skamnaki, V. T.; Osz, E.; Szilagyi, L.; Somsak, L.; Docsa, T.; Toth, B.; Gergely, P. *Bioorg. Med. Chem*. **2002**, *10*, 261–268 and Cecioni, S.; Argintaru, O.-A.; Docsa, T.; Gergely, P.; Praly, J.-P.; Vidal, S. *New J. Chem*., **2009**, *33*, 148-156.)

Glycogen phosphorylase *b* was prepared from rabbit skeletal muscle according to the method of Fischer and Krebs, (Fischer, E. H.; Krebs, E. G. *Methods Enzymol*. **1962**, *5*, 369–373) using dithiothreitol instead of L-cysteine, and recrystallized at least three times before use with a specific activity of 55 U/mg protein. Kinetic data for the inhibition of rabbit skeletal muscle glycogen phosphorylase were collected using different concentrations of α-D-glucose-1-phosphate (2 and 20 mM), constant concentrations of glycogen (1% w/v) and AMP (1 mM), and various concentrations of inhibitor. Inhibitor was dissolved in dimethyl sulfoxide (DMSO) and diluted in the assay buffer (50 mM triethanolamine, 1 mM EDTA and 1 mM dithiothreitol) so that the DMSO concentration in the assay should be lower than 1%. The enzymatic activities were presented in the form of doublereciprocal plots (Lineweaver–Burk) applying a nonlinear data analysis program. The means of standard errors for all calculated kinetic parameters averaged to less than 10%. IC_{50} values were determined in the presence of 4 mM α-D-glucose-1-phosphate, 1 mM AMP, 1% glycogen, and varying concentrations of the inhibitor.

The stock solution of GP inhibitor was made by DMSO as a solvent and the concentration was 250 mM. The final concentrations of the compounds in the assay were 6.25, 12.5, 31.25, 62.5, 125, 312.5 and 625 μM (this is the highest because the DMSO concentration was 0.25% in the system and has no effect on the enzyme activity).
